# Titanium-Mediated Reduction of Carboxamides to Amines with Borane–Ammonia

**DOI:** 10.3390/molecules28124575

**Published:** 2023-06-06

**Authors:** P. Veeraraghavan Ramachandran, Abdulkhaliq A. Alawaed, Aman Singh

**Affiliations:** Department of Chemistry, Purdue University, 560 Oval Drive, West Lafayette, IN 47907, USA

**Keywords:** deoxygenation, borane–ammonia, carboxamides, amines, titanium tetrachloride, catalysis

## Abstract

In this study, the successful titanium tetrachloride-catalyzed reduction of aldehydes, ketones, carboxylic acids, and nitriles with borane–ammonia was extended to the reduction (deoxygenation) of a variety of aromatic and aliphatic pri-, sec- and tert-carboxamides, by changing the stoichiometry of the catalyst and reductant. The corresponding amines were isolated in good to excellent yields, following a simple acid–base workup.

## 1. Introduction

The reduction of a carbonyl moiety is an extremely important transformation that occurs during organic syntheses [1]. While aldehydes, ketones, carboxylic acids, and esters provide the product alcohols, the reduction of carboxamides typically generates deoxygenated amines [2,3], an extremely important class of compounds due to their multifaceted functions [4,5,6]. Although they are efficient for carboxamide reduction, typical hydride reducing agents such as lithium aluminum hydride [7] are hazardous due to their air and moisture sensitivity. On the other hand, sodium borohydride (SBH) can reduce amides only in the presence of activators [8,9,10,11,12,13] or heat [14,15]. The activators are presumed to generate borane in situ or undergo a cation exchange. The reduction can also be achieved by changing the cation of the metal borohydride [16,17]. Lewis acidic borane reagents such as diborane [18], borane-tetrahydrofuran (B-THF) [19], and borane–dimethyl sulfide (BMS) [20,21] readily reduce amides to amines. However, these extremely sensitive reagents also require anhydrous conditions during their reactions. Metal-catalyzed [22,23,24,25,26] or thermal [27] reductions of amides using borane derivatives, such as pinacolborane, have also been reported. Less bulky dialkylboranes such as 9-borabicyclo[3.3.1]nonane (9-BBN) also reduce tert-amides to amines at room temperature, whereas bulky dialkylboranes, such as disiamylborane and dicyclohexylborane reduce them to alcohols [28].

The reduction of amides with hydrosilanes has been examined over several decades with a large number of metal (B, Cs, Al, Zn, Au, Re, Os, Ru, Rh, Ti, Mo, Mn, Fe, Co, Ni, Cu, In, Ir, Pd, Pt, and La, etc.) catalysts [3]. The metal-free [2] reduction of amides with hydrosilanes has also been reported.

The hydrogenative deoxygenation of carboxamides with molecular hydrogen in the presence of a variety of catalysts has also been reported [3]. However, the lack of chemoselectivity of this process is a major drawback that prevents its utility.

In comparison, only sparse examples of carboxamide reduction with borane–amines have been reported. Although they are air- and moisture-stable and safe to handle in open-air environments, their strong complexation with amines renders the borane–amines less reactive. The weak coordination between boron and nitrogen in bulky trialkylamine–boranes was exploited for representative tert-amide reduction using *N*,*N*-diethylaniline borane [29,30]. The reduction of tert-carboxamides and lactams using aminoborohydrides [28,31], as well as sec- and tert-amides using aminodiborane [32], both generated from borane–amines, have also been reported. A recent report described the deoxygenative reduction of carboxamides with borane–ammonia (**1a**) [33], wherein the amide and excess (4 equiv) **1a**, catalyzed by trispentafluorophenylborane [(C_6_F_5_)_3_B] and boron trifluoride etherate (BF_3_-Et_2_O) (co-catalyst), were refluxed in 1,2-dichloroethane (DCE) for 24 h. Both catalysts are necessary for effective reduction. Boron trifluoride (0.3 equiv) was reported to activate the carbonyl moiety of the amide for the (C_6_F_5_)_3_B-catalyzed reduction (Equation (1)). Considerable deamination of the amide was observed if the stoichiometry of boron trifluoride was decreased.
(1)
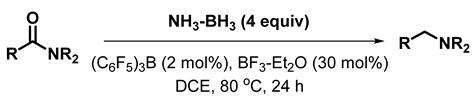



The reduction of amides using aminodiborane, generated in situ from borane–ammonia and molecular iodine (30–100 mol%), according to our recent report [34], is also carried out in refluxing DCE (Equation (2)) [32]. Once again, use of excess (4 equiv) **1a** and 30–100 mol% of iodine are critical for this aminodiborane-mediated deoxygenative reduction protocol.
(2)
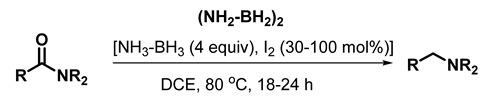



As part of our ongoing projects on the synthesis [35,36] and application of borane–amines [37], we recently reported the conversion of ketones [38] and carboxylic acids [39] to alcohols (Equation (3)) with borane–ammonia in the presence of titanium tetrachloride as an activator of the carbonyls. During the latter project, we had carried out a competitive reduction of an acid and a nitrile or an amide, and described the exclusive reaction of the acids. Further study involving the reduction of nitriles alone, by modifying the stoichiometry of the catalyst and reagents, led to an efficient reduction of nitriles to pri-amines (Equation (4)) [40]. Curious to learn whether an amide can also be reduced by varying the catalyst/reagent stoichiometry, a project was undertaken to expand our study to TiCl_4_-mediated reduction with borane–ammonia. The importance of amines in organic and medicinal chemistry provided the necessary impetus.
(3)
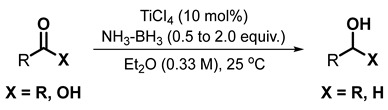

(4)
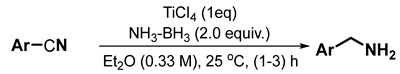



We reasoned that the lack of interest in borane–amines for the reduction of amides could be due to the possibility of contamination of product amines with the amine released from the borane–amine reagent. We envisioned that the use of borane–ammonia would release only ammonia and provide pure product amines. On the basis of the reported reduction of amides using 2 equivalents of borane derivatives, such as BTHF and BMS [18,19], we aimed to achieve the reduction, if possible, using ≤2.0 equivalents of borane ammonia, as was the case for the reduction of acids [39] and nitriles [40]. Accordingly, a systematic examination was initiated, and reported in this study is a facile process for the conversion of carboxamides to the corresponding amines with borane–ammonia (2 equiv) in the presence of 0.1–0.5 molar equivalents of titanium tetrachloride as the activator in refluxing DCE. Carboxamides derived from both aliphatic and aromatic carboxylic acids and amines underwent reduction, and the products were isolated in good to excellent yields, using a simple acid–base workup.

## 2. Results and Discussion

The rate of conversion of amides to amines is highly dependent on the nature and quantity of the Lewis acid used, the solvent, and the reaction temperature. The results of our optimization studies are summarized in Table 1. Attempting the reduction of a representative amide, *N*-benzylbenzamide (**2a**) with **1a**, under conditions identical to that for the carboxylic acid reduction [39] (10 mol% TiCl_4_ and 2 molar equiv of **1a** in diethyl ether at room temperature, 4 h), showed a 13% conversion rate to the product dibenzylamine, **3a**, as determined after a base workup of the initially formed hydrochloride salt. Indeed, the amine hydrochloride was the initial product in all of the reactions, and it is noteworthy that the hydrochloride salt was generated without the use of an external acid. Increasing the reaction time to 12 h showed a 20% conversion rate to product **3a** (Table 1, entry 2). Changing the solvent to tetrahydrofuran (THF) and reaction with 10% TiCl_4_ for 12 h showed no reaction (entry 3), presumably due to the complexation of the catalyst with the solvent. Increasing the catalyst load to 50 mol% also yielded similar results (entry 4); when the reaction was heated to reflux for 12 h, the NMR of the reaction mixture after base workup showed 73% conversion to **3a**, along with unreacted **2a** (entry 5). A similar reaction with 50 mol% TiCl_4_ in refluxing toluene and chloroform for 12 h also revealed 75% and 82% conversions, respectively (entries 6 and 7, respectively). These results suggested that the reaction temperature had more influence than the nature of the solvent, and that a higher mol% of catalyst loading is essential for the complete reduction to occur within a short time.

Noting that the earlier reductions [32,33] with **1a** were carried out in refluxing DCE, initially, a reaction with 10 mol% TiCl_4_ was attempted when we observed a complete reaction occurring within 24 h (entry 8). The base workup provided a 98% isolated yield of **3a**. The catalyst loading was increased to 20 mol% with refluxing for 18 h, yet expecting a faster reaction showed only a conversion of 94%, with 6% of unreacted **2a** (entry 9). Another reaction that was maintained under the same conditions for 24 h was complete, and the workup provided a 99% yield of **3a** (entry 10). Finally, a reaction of **2a** with a further increase to 0.5 molar equiv of TiCl_4_ in refluxing DCE was completed within 12 h, and **3a** was isolated with a 96% yield (entry 11). Clearly, increased catalyst loading accelerated the reactions, though their completion could be achieved with 10 mol% catalyst as well.

The effect of the catalyst was examined with other group IV catalysts, such as ZrCl_4_, and HfCl_4_, as well as with FeCl_3_ (entries 12–14). The conversion rates were very poor. Thus, a reaction in refluxing DCE with two equiv of **1a** and 50 mol% TiCl_4_ was chosen as the optimal conditions for subsequent studies (Equation (5)).
(5)
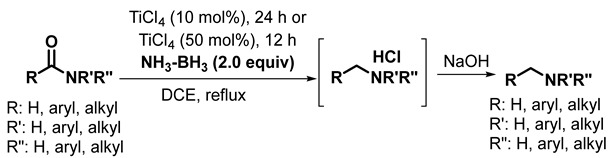



The scope and limitations of this TiCl_4_-mediated reduction of amides with borane–ammonia in refluxing DCE were next probed with respect to the carboxamide partners (Table 2 and Table 3). A representative series of carboxamides, prepared from a combination of both aryl and aliphatic carboxylic acids, with both aryl and alkyl amines, were reduced in the presence of 50 mol% TiCl_4_. Although the reactions were slower, representative examples of amides were also reduced in the presence of 10 mol% of TiCl_4_.

The aryl group in the acyl moiety was decorated with electron-donating and withdrawing groups (Table 2). Using electron-donating -OMe (**2b**) and -CH_3_ (**2c**) in the para position led to the corresponding dibenzyl amines (**3b** and **3c**, respectively) in similar yields (95%). The reaction of the amide with an electron-withdrawing CF_3_ group (**2d**) was much slower. The workup after 12 h revealed a mixture of the desired amine **3d**, along with unreacted **2d**. An acid–base workup separated the amine **3d**, isolated at a 65% yield. The reduction of *N*-phenylbenzamide (**2e**), where the benzyl group of the amine is replaced with a phenyl group, provided the corresponding amine **3e** at a good yield (88%) with 10 mol% TiCl_4_, within 12 h. Another reaction with 50 mol% catalyst provided 94% of **3e** in 12 h. Similarly, *N*-cyclohexylbenzamide (**2f**) produced 91% and 97% yields of the amine **3f** with 10 mol% and 50 mol% catalysts, within 24 h and 12 h, respectively. Morpholino(phenyl)methanone (*N*-benzoylmorpholine, **2g**) and *N*,*N*-dibenzylbenazmide (**2h**) were reduced within 12 h using 50 mol% TiCl_4_ to the corresponding amines, **3g** and **3h**, in 95% and 85% yields, respectively.

The reaction was equally effective for aliphatic amides as well (Table 3). *n*-Hexanamide (**2i**), a representative primary amine, was converted to the corresponding amine hydrochloride salt at an 80% yield with 50 mol% of the catalyst. Due to the water solubility of the amine, product **3i** was separated as a salt itself. *N*-Benzylformamide (**2j**) and *N*-phenylacetamide (**2k**) were both reduced to the corresponding amines **3j** and **3k** in 95% yields. All of the other aliphatic amides (**2l**–**2o**) examined provided the amines (**3l**–**3o**) in 90–98% yields. An aliphatic cyclic amide, caprolactam (**2p**), was reduced within 12 h to azepane (**3p**) in 85% yield, again isolated as the hydrochloride salt due to the solubility of the amine in water.

It is noteworthy that compared to the earlier reported reductions of amides using borane–ammonia, directly [33] or indirectly [32], utilized twice the amount of the reducing agent used in our protocol. Moreover, our reaction was completed in half the time, and all classes of amides (*pri*-, *sec*-, and *tert*-) were within the scope of the reduction. These properties make our protocol superior to those reported.

As reported earlier, carboxylic acids can be selectively reduced in the presence of an amide, using this titanium tetrachloride-catalyzed reaction with borane–ammonia [39]. It is known that an amide is less reactive with a hydride nucleophile than a ketone and ester moieties. We undertook examination of carbonyl group selectivity, and carried out the reduction of a 1:1 mixture of acetophenone and *N*-benzylbenzamide (**2a**) under the conditions for the ketone reduction [38] (10% TiCl_4_, 0.5 equiv of borane–ammonia in Et_2_O, RT, 1 h), and observed that the ketone was reduced completely to the alcohol in preference to the amide. None of the product amine **3a** was obtained after the workup. The same reaction in DCE as solvent was much slower. After 20 h at RT, ~6% of the ketone remained, and 2% of the product amine **3a** was also observed in the product mixture after the workup. However, the reduction performed under the conditions for the current amide reduction protocol (50% TiCl_4_, 2 equiv of borane–ammonia in refluxing DCE for 12 h) showed that the ketone was reduced completely, and 34% of the amide was also converted to the amine **3a** with 66% of the amide unreacted. This can be understood with the excess borane–ammonia present in the medium, relative to what is necessary for a complete ketone reduction.

A similar reduction of a 1:1 mixture of ethyl benzoate and *N*-benzylbenzamide (**2a**) under the conditions for the amide reduction showed no selectivity for either the ester or amide. The workup after 12 h showed 43% of alcohol and 34% of amine products, along with the unreacted starting materials.

## 3. Materials and Methods

### 3.1. General Information

The borane–ammonia was prepared according to our earlier published procedure [41]. The carboxamides used in this project were prepared using our borane-mediated amidation protocols [37,42]. The other reagents, solvents, carboxylic acids, as well as the amines used to prepare the carboxamides, were purchased from Sigma-Aldrich or Oakwood Chemical. The sodium borohydride and 1,2-dichloroethane were used as received.

Thin-layer chromatography (TLC) was performed on silica gel F60 plates and visualized under UV light or a ceric ammonium molybdate solution. The structures of the product amines were confirmed with nuclear magnetic resonance (NMR) spectroscopy, and measured in δ values in parts per million (ppm). The ^1^H, ^13^C, and ^19^F NMR spectra of the reduction products were recorded on a Bruker 400 MHz spectrometer at ambient temperature. The ^1^H spectra were calibrated against the residual solvent peak of CDCl_3_ (ẟ = 7.26 ppm) as an internal standard. The ^13^C NMR spectra were reported at 101 MHz and calibrated using CDCl_3_ (ẟ = 77.0 ppm) as an internal standard. The coupling constants (*J*) were given in hertz (Hz), and the signal multiplicities were described for the NMR data as s = singlet, d = doublet, t = triplet, dd = doublet of doublets, dt = doublet of triplets, qd = quartet of doublets, q = quartet, quint and p = pentet, m = multiplet, and br = broad. The ^11^B NMR spectra of the synthesized borane–ammonia was recorded at 96 MHz, and its chemical shifts were reported relative to the external standard, BF_3_-OEt_2_ (ẟ = 0 ppm), on a Varian INOVA or MERCURY 300 MHz NMR instrument. The ^19^F NMR spectra were recorded at 376 MHz and calibrated using CFCl_3_ (δ = 0 ppm) as the external standard.

### 3.2. Experimental

A description of the general procedure for the deoxygenation of carboxamides to amines follows. The preparation of dibenzylamine from *N*-benzylbenzamide was typical.

A 50 mL oven-dried round bottom flask with a sealed side arm was charged with *N*-benzylbenzamide (1.0 mmol) and a magnetic stirring bar. The flask was sealed using a rubber septum, and 1,2-dichloroethane (or other solvents) (10 mL) was added, followed by the dropwise addition of TiCl_4_ (or other Lewis acids) (0.1–0.5 equiv) via syringe if a liquid was involved, or by temporarily removing the septum if a solid was involved. Then, the septum was carefully opened, and ammonia borane (2.0 mmol, 2.0 equiv) was added slowly to the reaction mixture. A reflux condenser was attached to the flask, and the reaction mixture was brought to reflux using an oil bath, and monitored with TLC. After completion (~12 h), the reaction mixture was quenched with 3 *M* sodium hydroxide solution (3 mL), transferred to a separatory funnel, and extracted with dichloromethane (DCM) (3 × 2 mL). The organic layer was dried with sodium sulfate, filtered through cotton, and concentrated under aspirator vacuum using a rotary evaporator. Any remaining traces of solvent were removed by subjecting the solution to high vacuum for 30 min. The product amines were characterized using ^1^H and ^13^C NMR spectroscopy and compared with those reported in the literature (see Supporting Information).

The preparation of hexan-1-amine hydrochloride involved a similar procedure as mentioned above; after completion of the reaction (~12 h), the mixture was quenched with distilled water (1 mL), transferred to a separatory funnel, and extracted with DCM (3 × 2 mL). The organic layer was dried with sodium sulfate, filtered through cotton, and concentrated under aspirator vacuum using a rotary evaporator. Any remaining traces of solvent were removed by subjecting the solution to high vacuum for 30 min.

The preparation of azepane hydrochloride involved a similar procedure as mentioned above; after completion of the reaction (~12 h), the mixture was concentrated under aspirator vacuum using a rotary evaporator. Any remaining traces of solvent were removed by subjecting the solution to high vacuum for 30 min. The ^1^H and ^13^C NMR spectra were plotted for the crude product.

#### Characterization of Product Amines

*Dibenzylamine* (**3a**); The compound was prepared as described in the general procedure (yellow oil, mass = 189 mg, 96% yield); **^1^H NMR** (400 MHz, CDCl_3_) δ 7.4–7.3 (m, 8H), 7.3–7.2 (m, 2H), 3.8 (s, 4H). **^13^C NMR** (101 MHz, CDCl_3_) δ 140.2, 128.3, 128.1, 126.9, 53.1. The compound characterization is in accordance with previous reports [43].
*N-benzyl-1-(4-methoxyphenyl)methanamine* (**3b**); The compound was prepared as described in the general procedure (yellow oil, mass = 215 mg, 95% yield); **^1^H NMR** (400 MHz, CDCl3) δ 7.4–7.3 (m, 4H), 7.3–7.2 (m, 3H), 6.9–6.8 (m, 2H), 3.8 (s, 5H), 3.8 (s, 2H), 2.3 (s, 1H). **^13^C NMR** (101 MHz, CDCl3) δ 158.6, 139.9, 131.9, 129.4, 128.3, 128.2, 126.9, 113.7, 55.2, 52.8, 52.3. The compound characterization is in accordance with previous reports [43].
*N-benzyl-1-(p-tolyl)methanamine* (**3c**); The compound was prepared as described in the general procedure (yellow oil, mass = 200 mg, 95% yield); **^1^H NMR** (400 MHz, CDCl_3_) δ 7.4–7.3 (m, 4H), 7.3–7.2 (m, 3H), 7.2 (d, *J* = 7.9 Hz, 2H), 3.8 (s, 2H), 3.8 (s, 2H), 2.4 (s, 3H), 1.8 (s, 1H). **^13^C NMR** (101 MHz, CDCl_3_) δ 140.3, 137.2, 136.4, 129.0, 128.3, 128.1, 128.0, 126.8, 53.0, 52.8, 21.0. The compound characterization is in accordance with previous reports [43].
*N-benzyl-1-(4-(trifluoromethyl)phenyl)methanamine* (**3d**); The compound was prepared as described in the general procedure (colorless oil, mass = 172 mg, 65% yield); **^1^H NMR** (400 MHz, CDCl_3_) δ 7.6 (d, *J* = 8.0 Hz, 2H), 7.5 (d, *J* = 8.0 Hz, 2H), 7.4–7.2 (m, 5H), 3.9 (s, 2H), 3.8 (s, 2H), 1.9 (s, 1H).**^13^C NMR** (101 MHz, CDCl_3_) δ 144.3, 139.8, 129.3, 129.0, 128.4, 128.2, 128.1, 127.0, 125.23, 125.19, 53.1, 52.4. **^19^F NMR** (376 MHz, CDCl_3_) δ −63.8. The compound characterization is in accordance with previous reports [44].
*N-benzylaniline* (**3e**); The compound was prepared as described in the general procedure (colorless oil, mass = 172 mg, 94% yield); **^1^H NMR** (400 MHz, CDCl3) δ 7.5–7.3 (m, 4H), 7.3–7.3 (m, 1H), 7.2 (dd, *J* = 8.6, 7.3 Hz, 2H), 6.7 (tt, *J* = 7.3, 1.1 Hz, 1H), 6.7 (dd, *J* = 8.7, 1.1 Hz, 2H), 4.3 (s, 2H), 4.1 (s, 1H). **^13^C NMR** (101 MHz, CDCl3) δ 148.0, 139.3, 129.2, 128.5, 127.4, 127.1, 117.5, 112.77, 48.3. The compound characterization is in accordance with previous reports [45].
*N-benzylcyclohexanamine* (**3f**); The compound was prepared as described in the general procedure (yellow oil, mass = 182 mg, 97% yield); **^1^H NMR** (400 MHz, CDCl_3_) δ 7.3 (d, *J* = 4.4 Hz, 4H), 7.3–7.2 (m, 1H), 3.8 (s, 2H), 2.6–2.4 (m,1), 1.9 (d, *J* = 12.0 Hz, 2H), 1.8–1.7 (m, 2H), 1.6–1.6 (m, 1H), 1.3–1.1 (m, 6H). **^13^C NMR** (101 MHz, CDCl_3_) δ 140.9, 128.3, 128.0, 126.7, 56.1, 51.0, 33.5, 26.1, 24.9. The compound characterization is in accordance with previous reports [46].
*4-benzylmorpholine* (**3g**); The compound was prepared as described in the general procedure (yellow oil, mass = 168 mg, 95% yield); **^1^H NMR** (400 MHz, CDCl_3_) δ 7.4–7.3 (m, 4H), 7.3–7.2 (m, 1H), 3.7–3.7 (m, 4H), 3.5 (s, 2H), 2.5–2.4 (m, 4H). **^13^C NMR** (101 MHz, CDCl_3_) δ 137.6, 129.1, 128.2, 127.1, 66.9, 63.4, 53.5. The compound characterization is in accordance with previous reports [46].
*Tribenzylamine* (**3h**); The compound was prepared as described in the general procedure (yellow solid, mass = 243 mg, 85% yield); **^1^H NMR** (400 MHz, CDCl_3_) δ 7.5–7.4 (m, 6H), 7.4–7.3 (m, 6H), 7.3–7.2 (m, 3H), 3.6 (d, *J* = 3.2 Hz, 6H). **^13^C NMR** (101 MHz, CDCl_3_) δ 139.6, 128.7, 128.1, 126.8, 57.9. The compound characterization is in accordance with previous reports [46].
*Hexan-1-amine hydrochloride* (**3i**); The compound was prepared as described in the general procedure (white solid, mass = 109 mg, 80% yield); **^1^H NMR** (400 MHz, CDCl3) δ 6.0 (s, 2H), 2.7 (p, *J* = 7.5 Hz, 2H), 1.7 (p, *J* = 7.5 Hz, 2H), 1.4–1.2 (m, 3H), 0.9–0.8 (m, 1H). **^13^C NMR** (101 MHz, CDCl3) δ 44.5, 31.2, 28.7, 26.4, 22.4, 13.8. The compound characterization is in accordance with previous reports [47].
*N-methyl-1-phenylmethanamine* (**3j**); The compound was prepared as described in the general procedure (yellow oil, mass = 109 mg, 90% yield); **^1^H NMR** (400 MHz, CDCl_3_) δ 7.4–7.2 (m, 5H), 3.7 (s, 2H), 2.5 (s, 3H), 1.5 (s, 1H). **^13^C NMR** (101 MHz, CDCl_3_) δ 140.1, 128.3, 128.1, 126.8, 56.0, 36.0. The compound characterization is in accordance with previous reports [46].
*N-ethylaniline* (**3k**); The compound was prepared as described in the general procedure (yellow oil, mass = 115 mg, 95% yield); **^1^H NMR** (400 MHz, CDCl3) δ 7.2 (dd, *J* = 8.6, 7.3 Hz, 2H), 6.7 (tt, *J* = 7.3, 1.1 Hz, 1H), 6.6 (d, *J* = 7.6 Hz, 2H), 3.6 (s, 1H), 3.2 (q, *J* = 7.1 Hz, 2H), 1.3 (t, *J* = 7.1 Hz, 3H). **^13^C NMR** (101 MHz, CDCl3) δ 148.3, 129.1, 117.1, 112.7, 38.4, 14.8. The compound characterization is in accordance with previous reports [48].
*N-benzylhexan-1-amine* (**3l**); The compound was prepared as described in the general procedure (yellow oil, mass = 172 mg, 90% yield); **^1^H NMR** (400 MHz, CDCl_3_) δ 7.3 (d, *J* = 4.7 Hz, 4H), 7.3–7.2 (m, 1H), 3.8 (s, 2H), 2.7–2.6 (m, 2H), 1.5 (q, *J* = 7.9 Hz, 2H), 1.3–1.2 (m, 6H), 0.9 (t, *J* = 6.7 Hz, 3H). **^13^C NMR** (101 MHz, CDCl_3_) δ 140.5, 128.3, 128.0, 126.7, 54.0, 49.5, 31.7, 30.0, 27.0, 22.5, 14.0. The compound characterization is in accordance with previous reports [49].
*N-(cyclohexylmethyl)hexan-1-amine* (**3m**); The compound was prepared as described in the general procedure (yellow oil, mass = 183 mg, 93% yield); **^1^H NMR** (400 MHz, CDCl_3_) δ 2.55 (t, *J* = 7.2 Hz, 2H), 2.4 (d, *J* = 6.7 Hz, 2H), 1.7–1.6 (m, 5H), 1.5–1.4 (m, 3H), 1.3–1.2 (m, 9H), 0.9–0.9 (m, 5H). **^13^C NMR** (101 MHz, CDCl_3_) δ 56.9, 50.2, 37.9, 31.7, 31.4, 30.1, 27.0, 26.6, 26.0, 22.5, 14.0. The compound characterization is in accordance with previous reports [50].
*4-(cyclohexylmethyl)morpholine* (**3n**); The compound was prepared as described in the general procedure (colorless oil, mass = 180 mg, 98% yield); **^1^H NMR** (400 MHz, CDCl3) δ 3.7 (t, *J* = 4.5 Hz, 4H), 2.5–2.3 (m, 4H), 2.1 (d, *J* = 7.2 Hz, 2H), 1.8 (d, *J* = 12.7 Hz, 2H), 1.7–1.6 (m, 3H), 1.6–1.4 (m, 1H), 1.3–1.1 (m, 3H), 0.9–0.8 (m, 2H). **^13^C NMR** (101 MHz, CDCl3) δ 66.7, 65.9, 54.0, 34.4, 31.7, 26.6, 26.0. The compound characterization is in accordance with previous reports [51].
*4-phenethylmorpholine* (**3o**); The compound was prepared as described in the general procedure (yellow oil, mass = 187 mg, 98% yield); **^1^H NMR** (400 MHz, CDCl3) δ 7.3–7.2 (m, 2H), 7.2–7.2 (m, 3H), 3.8–3.7 (m, 4H), 2.9–2.8 (m, 2H), 2.6–2.6 (m, 2H), 2.6–2.5 (m, 4H). **^13^C NMR** (101 MHz, CDCl3) δ 140.1, 128.6, 128.3, 126.0, 66.9, 60.8, 53.6, 33.2. The compound characterization is in accordance with previous reports [52].
*Azepane hydrochloride* (**3p**); The compound was prepared as described in the general procedure (yellow solid, mass = 114 mg, 85% yield); **^1^H NMR** (400 MHz, CDCl_3_) δ 9.6 (s, 2H), 3.2 (p, *J* = 5.1 Hz, 4H), 2.0–1.9 (m, 4H), 1.8–1.7 (m, 4H). **^13^C NMR** (101 MHz, CDCl_3_) δ 45.6, 26.6, 25.1. The compound characterization is in accordance with previous reports [53].

## 4. Conclusions

In conclusion, we have developed a simple protocol for the reduction of carboxamides to afford amines, with good to excellent yields, using borane–ammonia as the reductant in the presence of 0.1–0.5 molar equivalents of TiCl_4_ in refluxing 1,2-dichloroethane. The reaction took 24 h to complete when 0.1 molar equiv of TiCl_4_ was used, and could be accelerated to 12 h by increasing the catalyst to 0.5 molar equiv. A broad range of aromatic, heteroaromatic, benzylic, and aliphatic amides were efficiently reduced under these conditions, in moderate to very high yields. This reducing system yields negligible side products, and the workup of the reaction mixture is very simple. The reaction is believed to progress via the activation of the carbonyl moiety of the amide by titanium tetrachloride, followed by the reduction with borane.

## Figures and Tables

**Table 1:** Reaction optimization for catalyzed reduction of *N*-benzylbenzamide ^a^.

Entry	LA	LA (mol%)	Solvent	Temp.	Time (h)	Product Conversion (%) ^b^
1	TiCl_4_	10	Et_2_O	RT	4	13
2	TiCl_4_	10	Et_2_O	RT	12	20
3	TiCl_4_	10	THF	RT	12	NR
4	TiCl_4_	50	THF	RT	12	NR
5	TiCl_4_	50	THF	reflux	12	73
6	TiCl_4_	50	Toluene	reflux	12	75
7	TiCl_4_	50	CHCl_3_	reflux	12	82
8	TiCl_4_	10	DCE	reflux	24	98 ^c^
9	TiCl_4_	20	DCE	reflux	18	94
10	TiCl_4_	20	DCE	reflux	24	99 ^c^
11	TiCl_4_	50	DCE	reflux	12	96 ^c^
12	ZrCl_4_	50	DCE	reflux	12	25
13	HfCl_4_	50	DCE	reflux	12	31
14	FeCl_3_	50	DCE	reflux	12	30

^a^ Reaction at appropriate temperature with two equiv of **1a**. ^b^ Incomplete reaction; conversion determined by PMR after base workup. ^c^ 100% conversion, isolated yield of **3a**.

**Table 2:** TiCl_4_-catalyzed reduction of aromatic carboxamides ^a^ with 2 equiv of borane–ammonia in refluxing DCE.

Entry	Amide		LA (mol%)	React. Time (h)	Product Amine		
	#	Structure			#	Structure	Yield (%) ^b^
1	**2a**	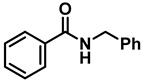	10	24	**3a**	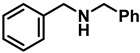	98
2	**2a**	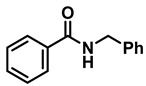	50	12	**3a**	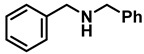	96
3	**2b**	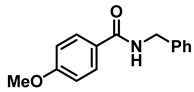	50	12	**3b**	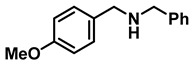	95
4	**2c**	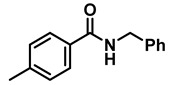	50	12	**3c**	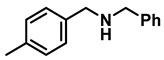	95
5	**2d**	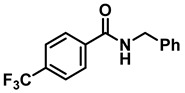	50	12	**3d**	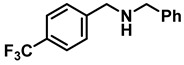	65 ^c^
6	**2e**	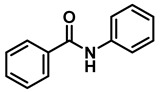	10	24	**3e**	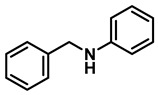	88
7	**2e**	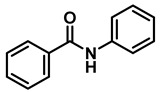	50	12	**3e**	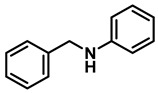	94
8	**2f**	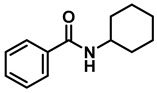	10	24	**3f**	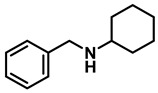	91
9	**2f**	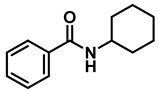	50	12	**3f**	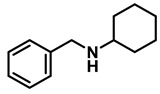	97
10	**2g**	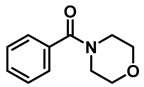	50	12	**3g**	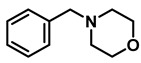	95
11	**2h**	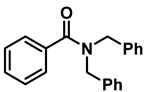	50	12	**3h**	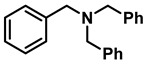	85

^a^ Carboxamides from aromatic acids. ^b^ Isolated yields of pure amines. ^c^ Incomplete reaction after 12 h. Yield of isolated amine.

**Table 3:** TiCl_4_-catalyzed reduction of aliphatic carboxamides ^a^ with two of equiv borane–ammonia.

Entry	Amide		LA (mol%)	React. Time (h)	Product Amine		
	#	Structure			#	Structure	Yield (%) ^b^
1	**2i**	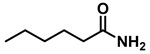	50	12	**3i**	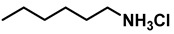	80
2	**2j**	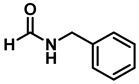	50	12	**3j**	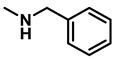	95
3	**2k**	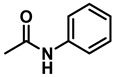	50	12	**3k**	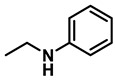	95
4	**2l**	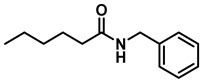	50	12	**3l**	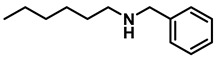	90
5	**2m**	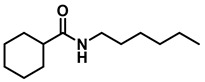	50	12	**3m**	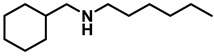	93
6	**2n**	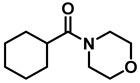	10	24	**3n**	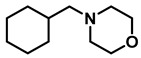	98
7	**2o**	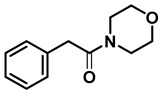	50	12	**3o**	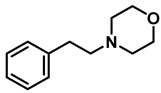	98
8	**2p**	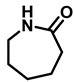	50	12	**3p**	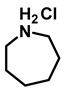	85

^a^ Carboxamides from aliphatic acids. ^b^ Isolated yield of pure amine or amine hydrochloride.

## Data Availability

All of the ^1^H and ^13^C NMR spectra are available in the Supporting Information.

## Supplementary Materials

The following supporting information can be downloaded at: https://www.mdpi.com/article/10.3390/molecules28124575/s1, NMR spectra of product amines.
