# Supplementary material for: Titanium-Mediated Reduction of Carboxamides to Amines with Borane–Ammonia
Source: Molecules. 2023 Jun 6;28(12):4575. doi: 10.3390/molecules28124575 (PMC10301125; doi:10.3390/molecules28124575)
Supplement: Supplementary file 1 [file molecules-28-04575-s001.zip › molecules-2426737-supplementary.pdf]

SUPPORTING INFORMATION

Titanium-Mediated Reduction of Carboxamides to Amines with Borane–Ammonia

P. Veeraraghavan Ramachandran,* Abdulkhaliq A. Alawaed, and Aman Singh

Department of Chemistry, Purdue University, 560 Oval Drive, West Lafayette, IN 47907, USA

E-mail: chandran@purdue.edu

Contents:

Page

NMR spectra of product amines S2-S18

<u>**NMR spectra of product amines**</u>

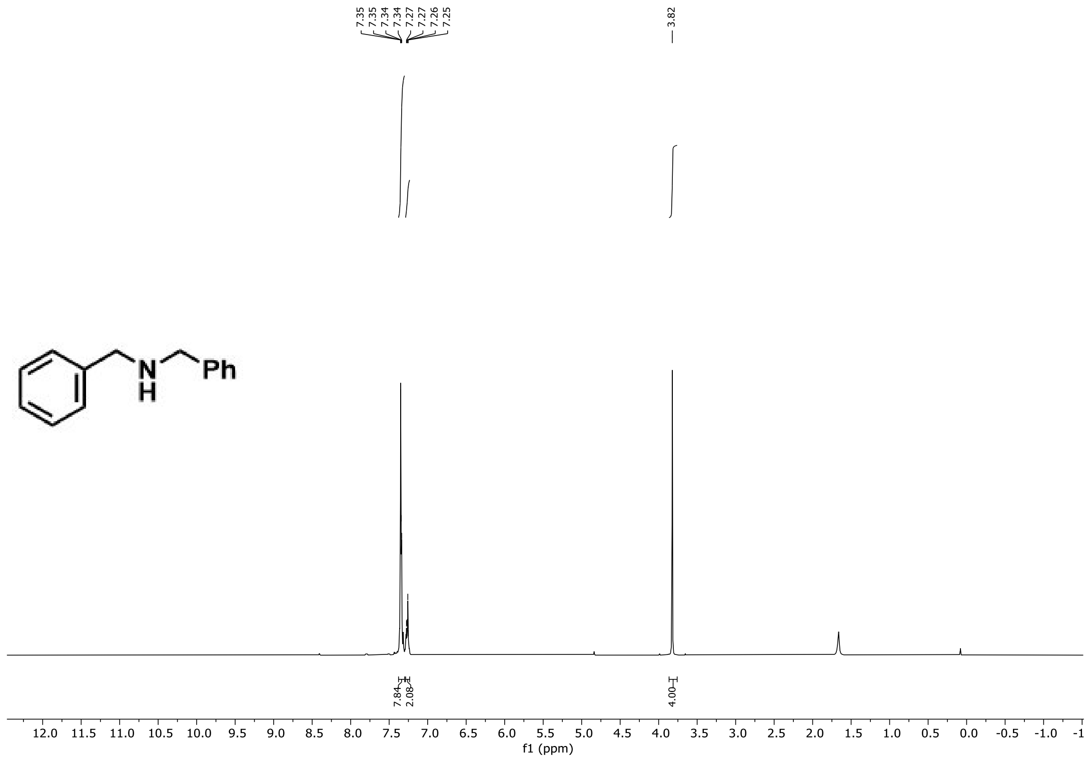

^{1}H NMR (400 MHz, $CDCl_3$) Dibenzylamine (**3a**)

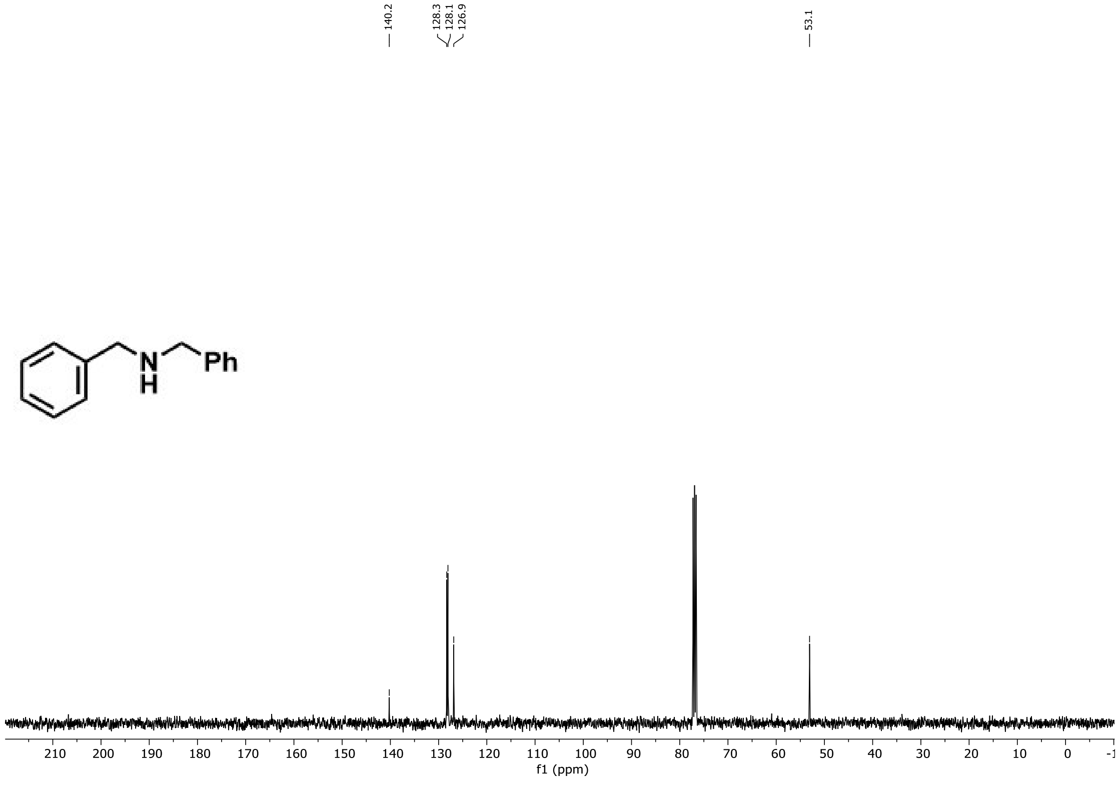

^{13}C NMR (101 MHz, $CDCl_3$) Dibenzylamine (**3a**)

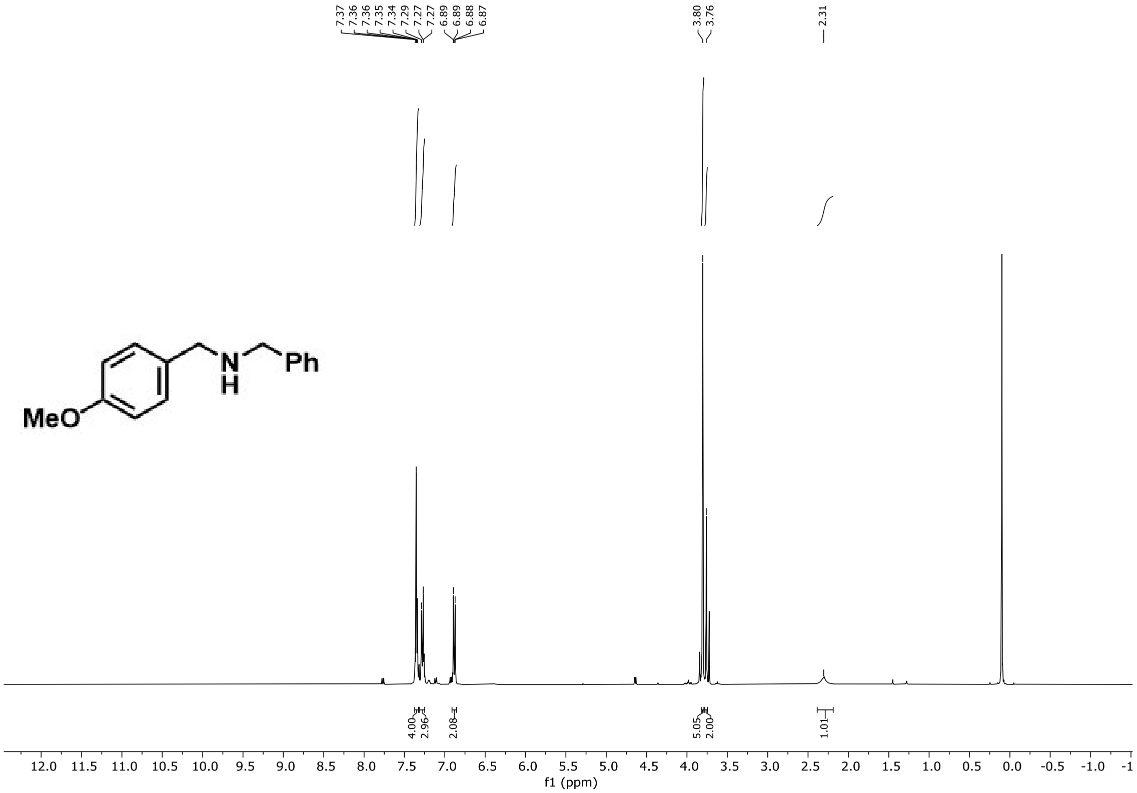

^{1}H NMR (400 MHz, $CDCl_3$) *N*-benzyl-1-(4-methoxyphenyl)methanamine (**3b**)

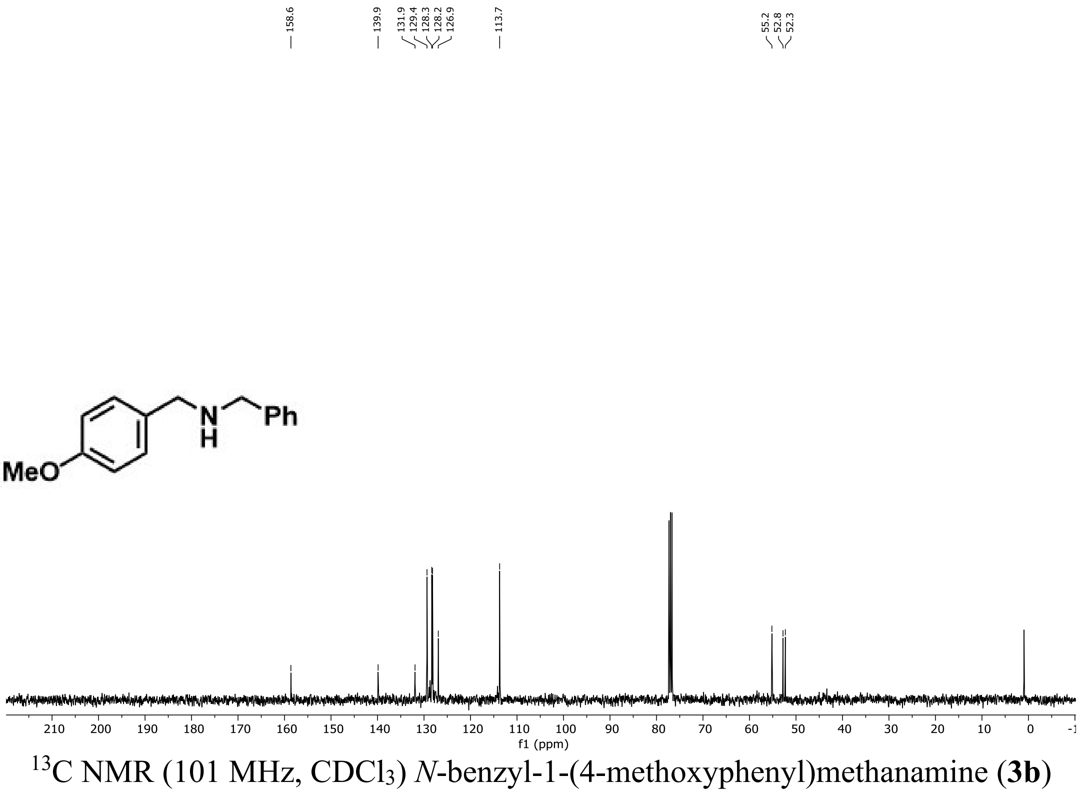

^{13}C NMR (101 MHz, $CDCl_3$) *N*-benzyl-1-(4-methoxyphenyl)methanamine (**3b**)

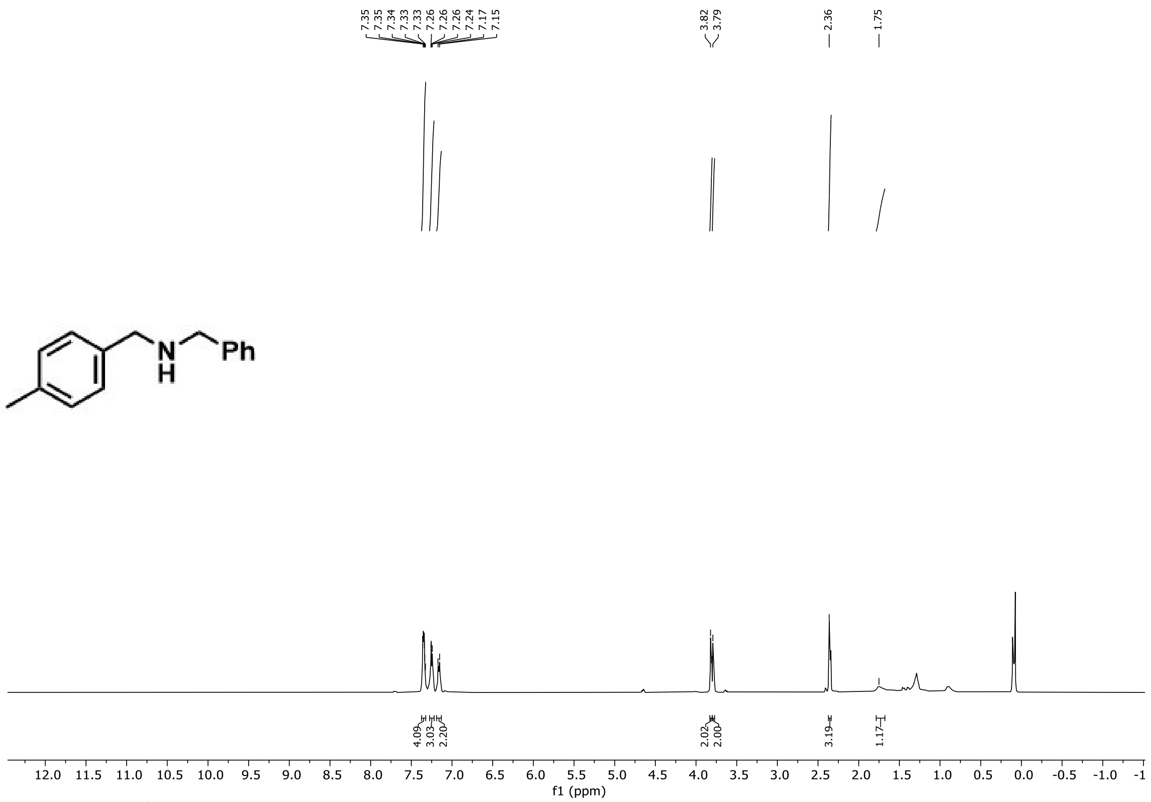

^{1}H NMR (400 MHz, $CDCl_3$) *N*-benzyl-1-(p-tolyl)methanamine (**3c**)

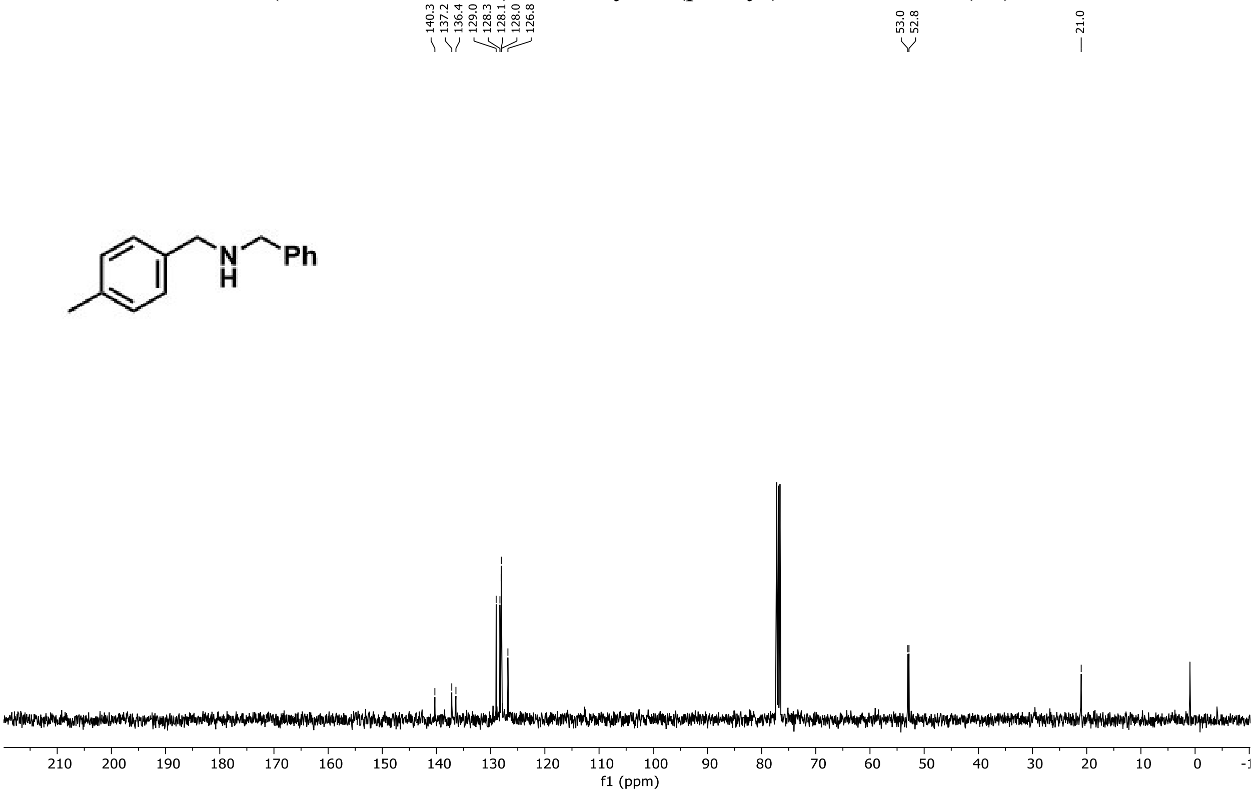

^{13}C NMR (101 MHz, $CDCl_3$) *N*-benzyl-1-(p-tolyl)methanamine (**3c**)

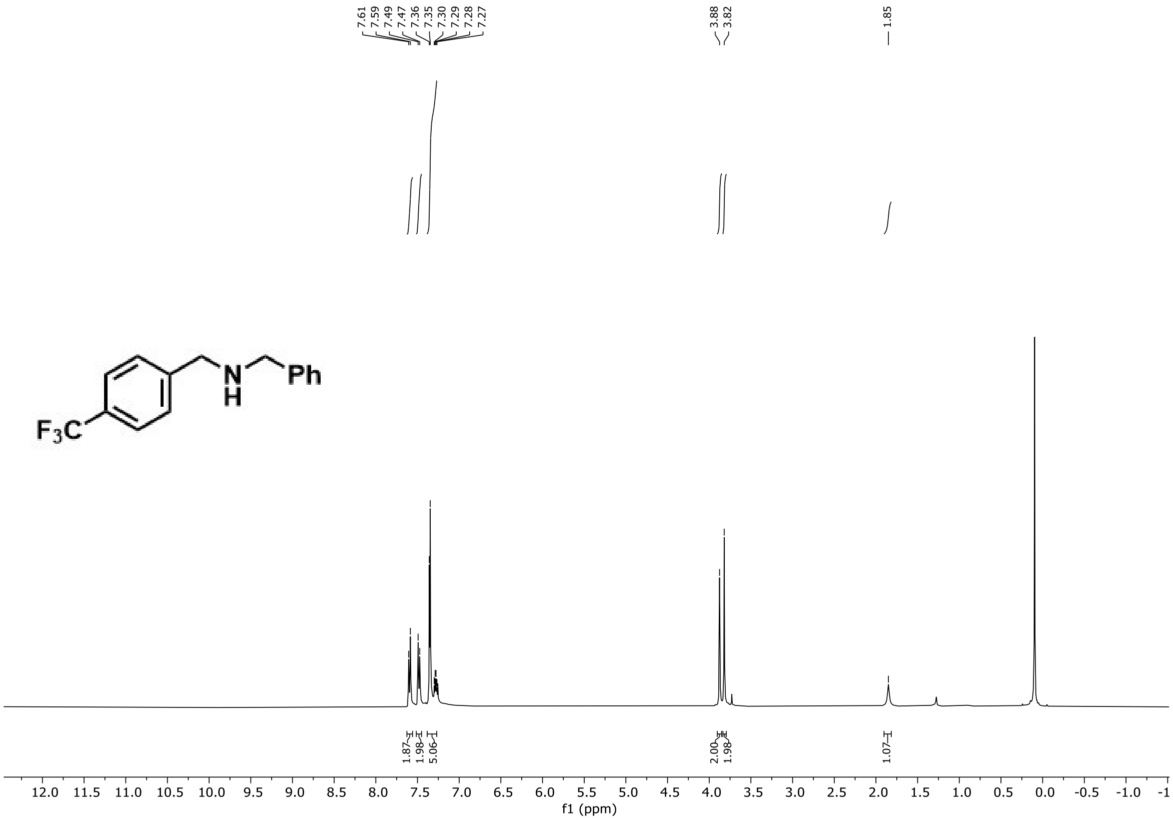

^{1}H NMR (400 MHz, $CDCl_3$) *N*-benzyl-1-(4-(trifluoromethyl)phenyl)methanamine (**3d**)

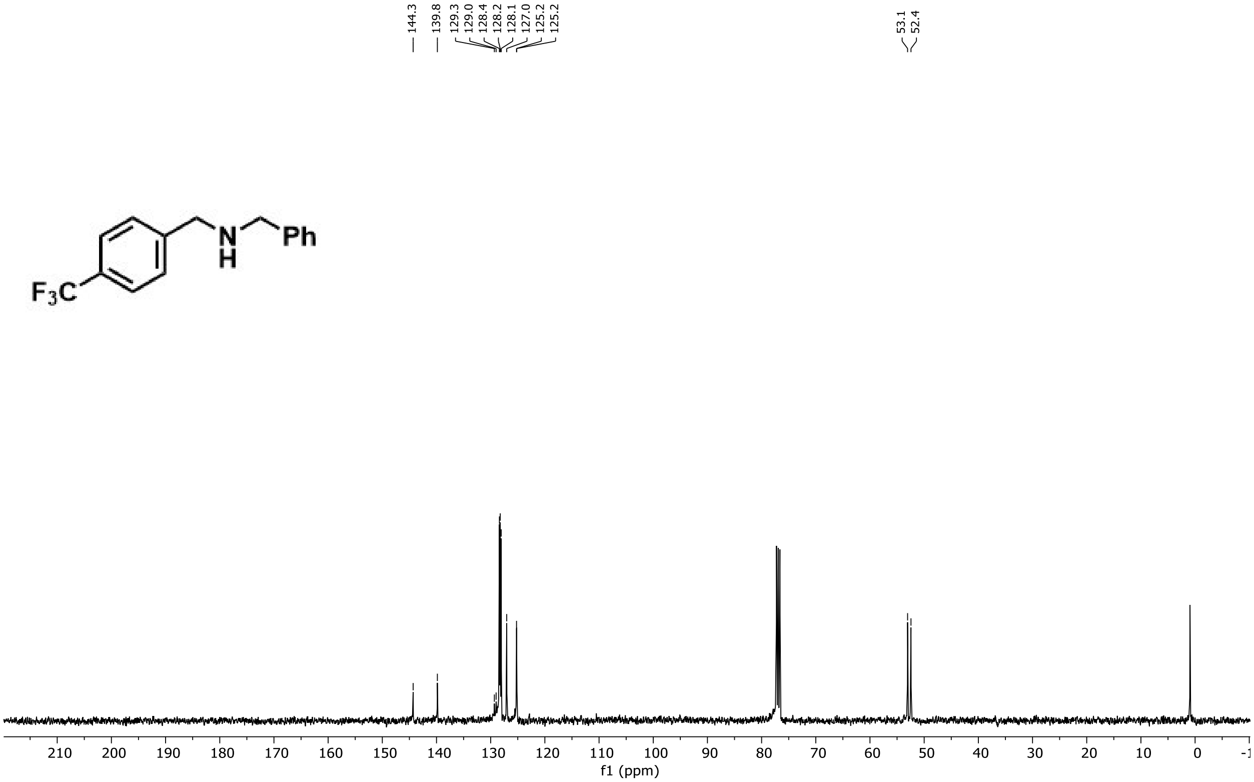

^{13}C NMR (101 MHz, $CDCl_3$) *N*-benzyl-1-(4-(trifluoromethyl)phenyl)methanamine (**3d**)

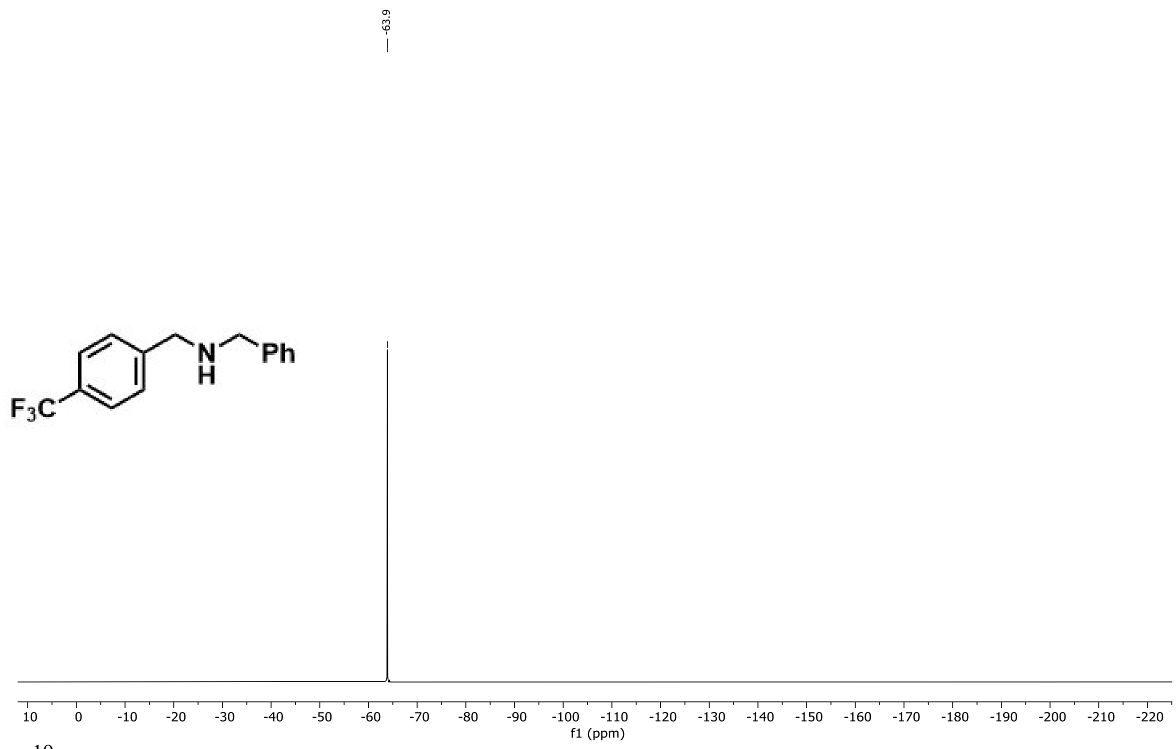

^{19}F NMR (376 MHz, $CDCl_3$) *N*-benzyl-1-(4-(trifluoromethyl)phenyl)methanamine (**3d**)

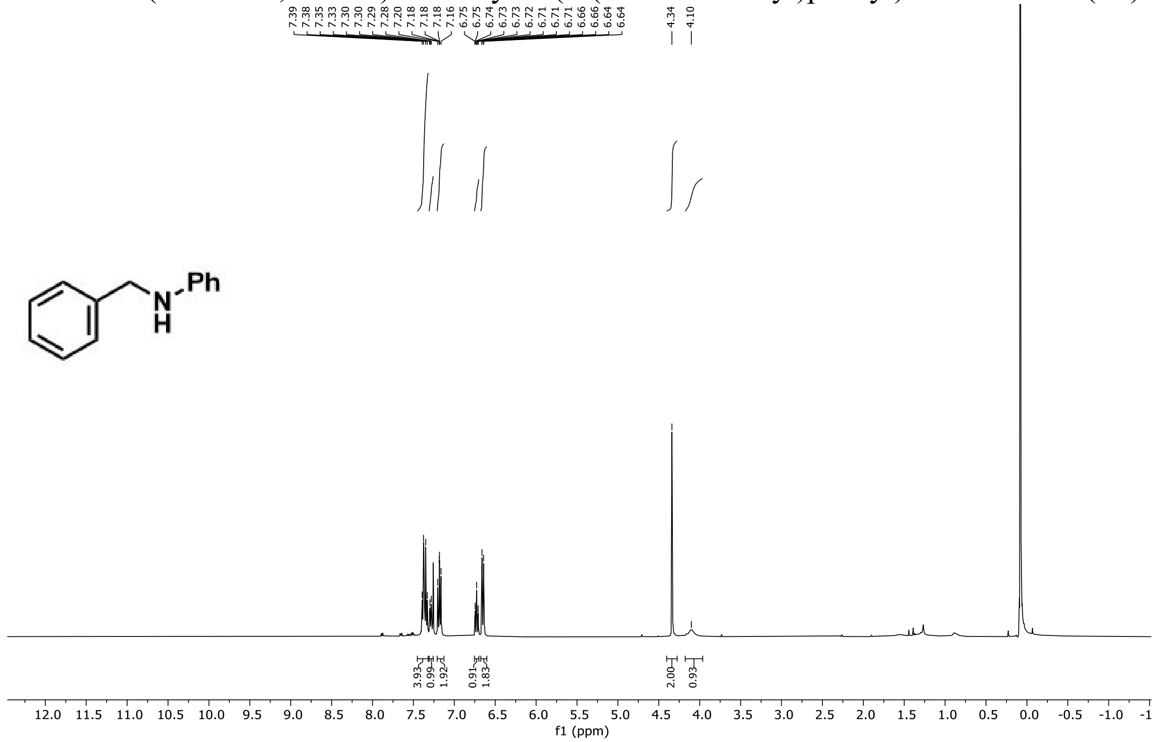

^{1}H NMR (400 MHz, $CDCl_3$) *N*-benzylaniline (**3e**)

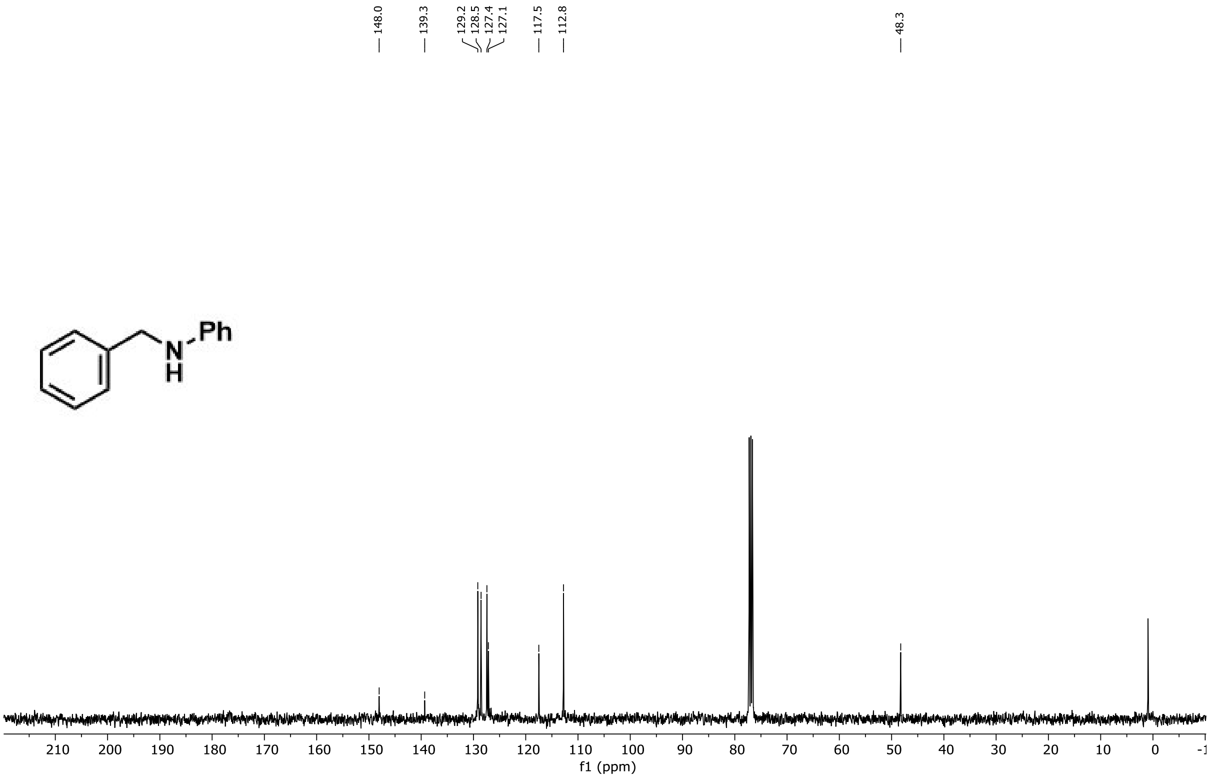

^{13}C NMR (101 MHz, $CDCl_3$) *N*-benzylaniline (**3e**)

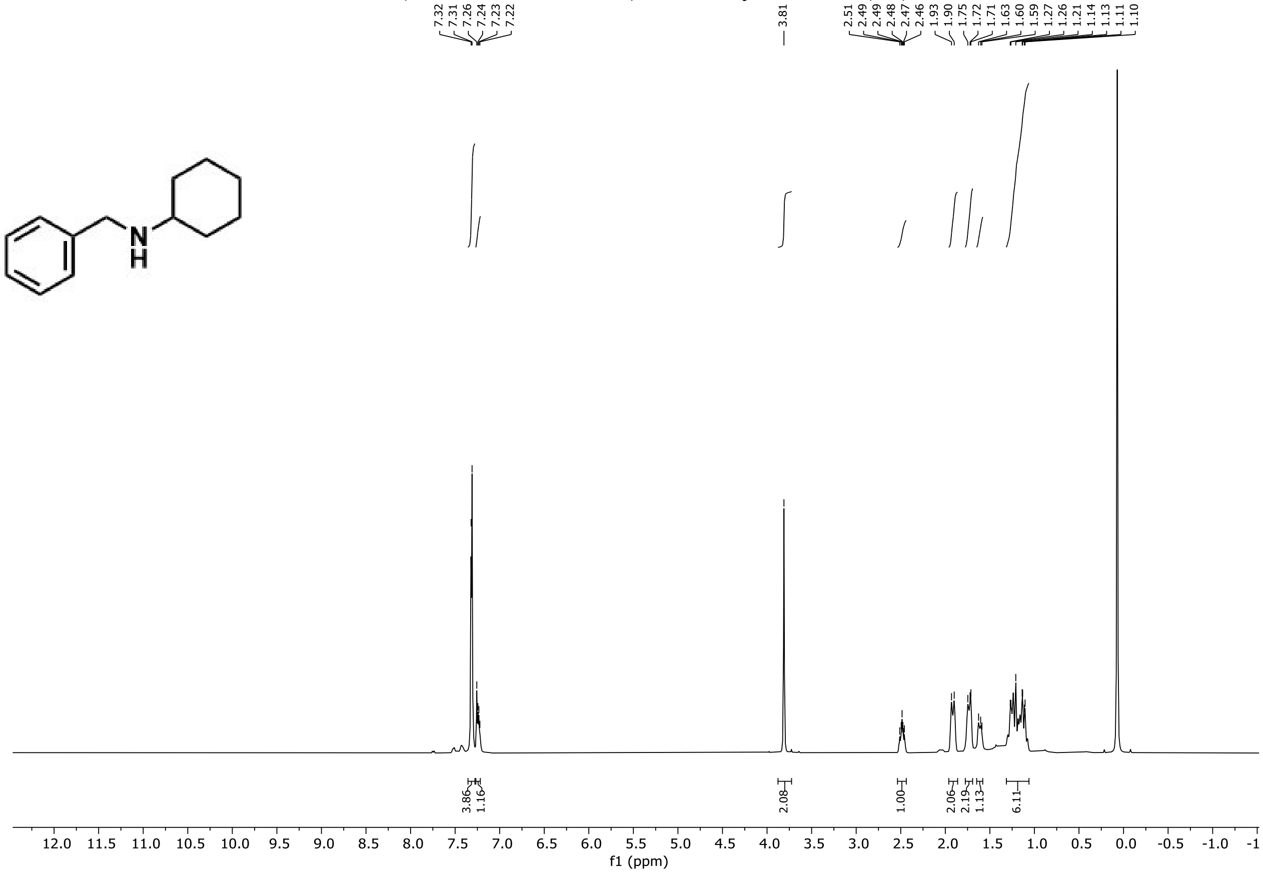

^{1}H NMR (400 MHz, $CDCl_3$) *N*-benzylcyclohexanamine (**3f**)

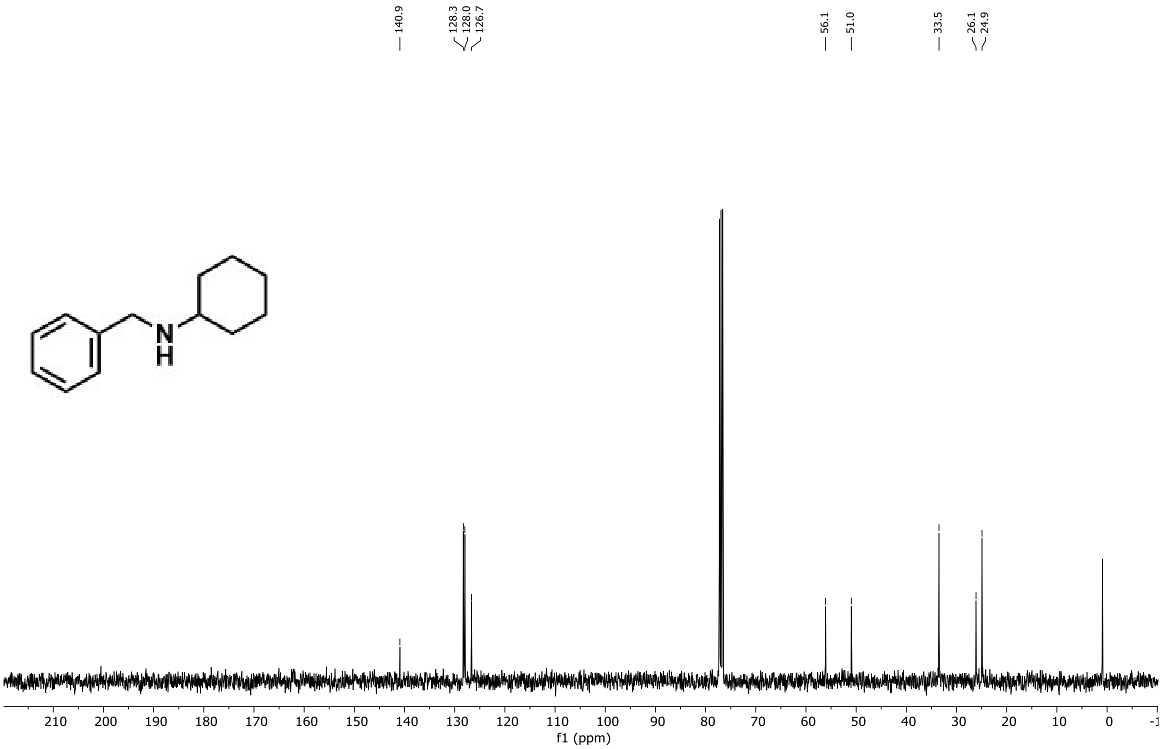

^{13}C NMR (101 MHz, $CDCl_3$) *N*-benzylcyclohexanamine (**3f**)

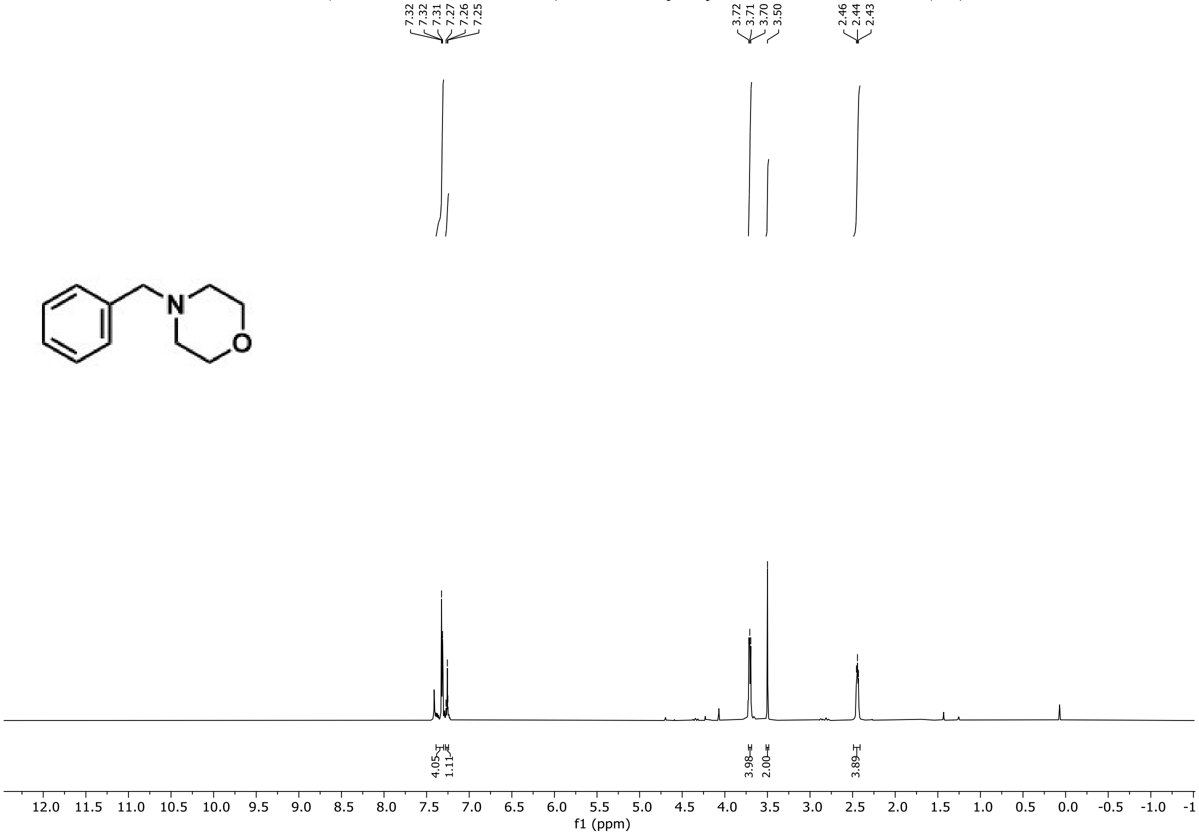

^{1}H NMR (400 MHz, $CDCl_3$) 4-benzylmorpholine (**3g**)

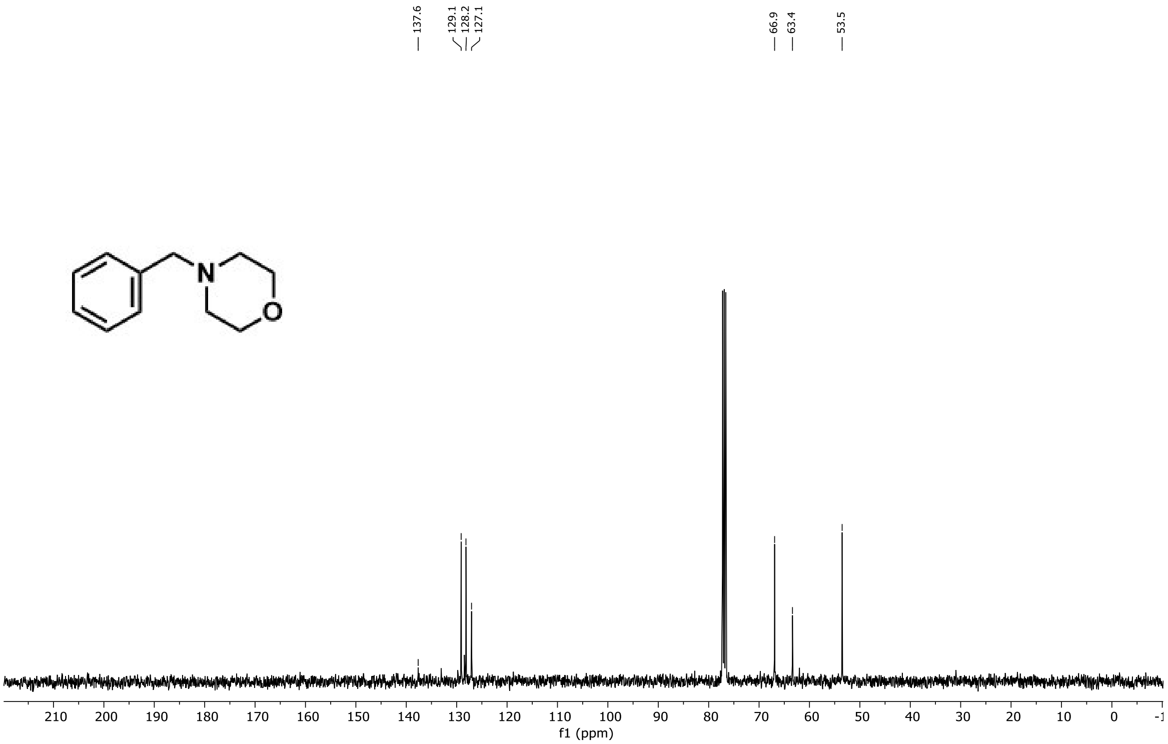

^{13}C NMR (101 MHz, $CDCl_3$) 4-benzylmorpholine (**3g**)

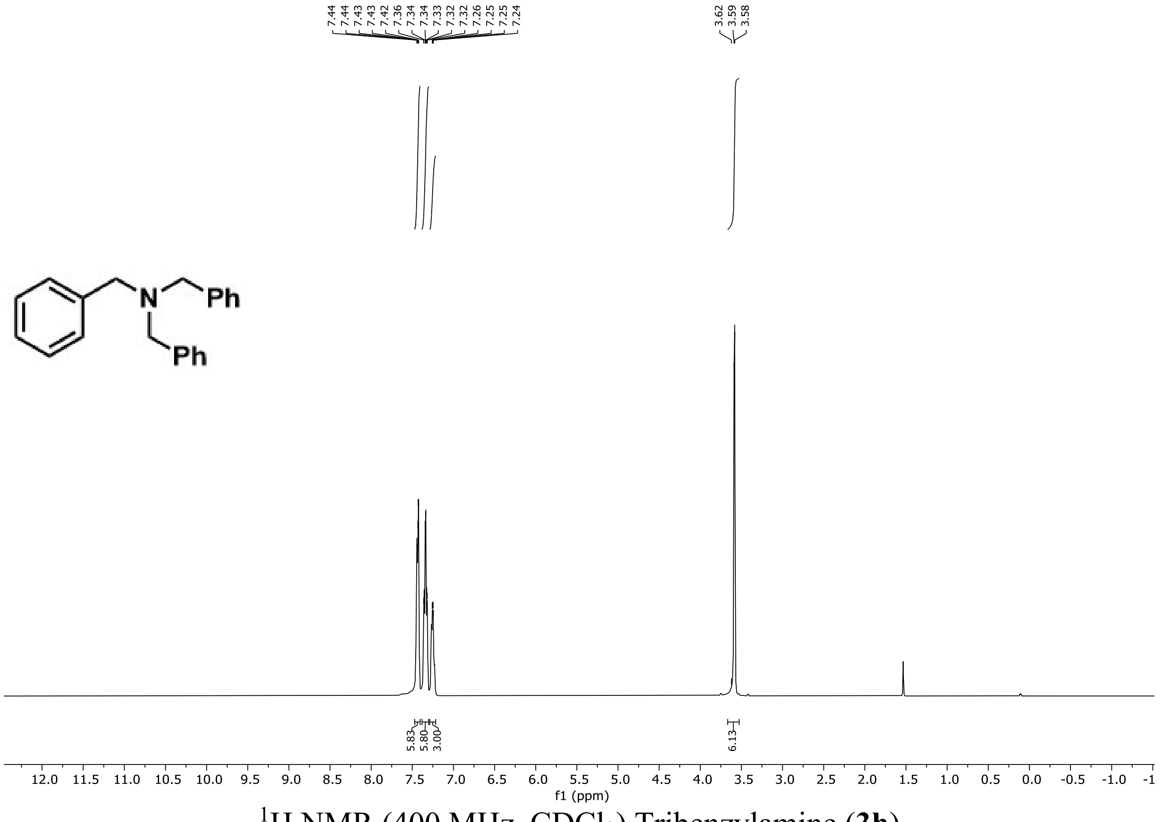

^{1}H NMR (400 MHz, $CDCl_3$) Tribenzylamine (**3h**)

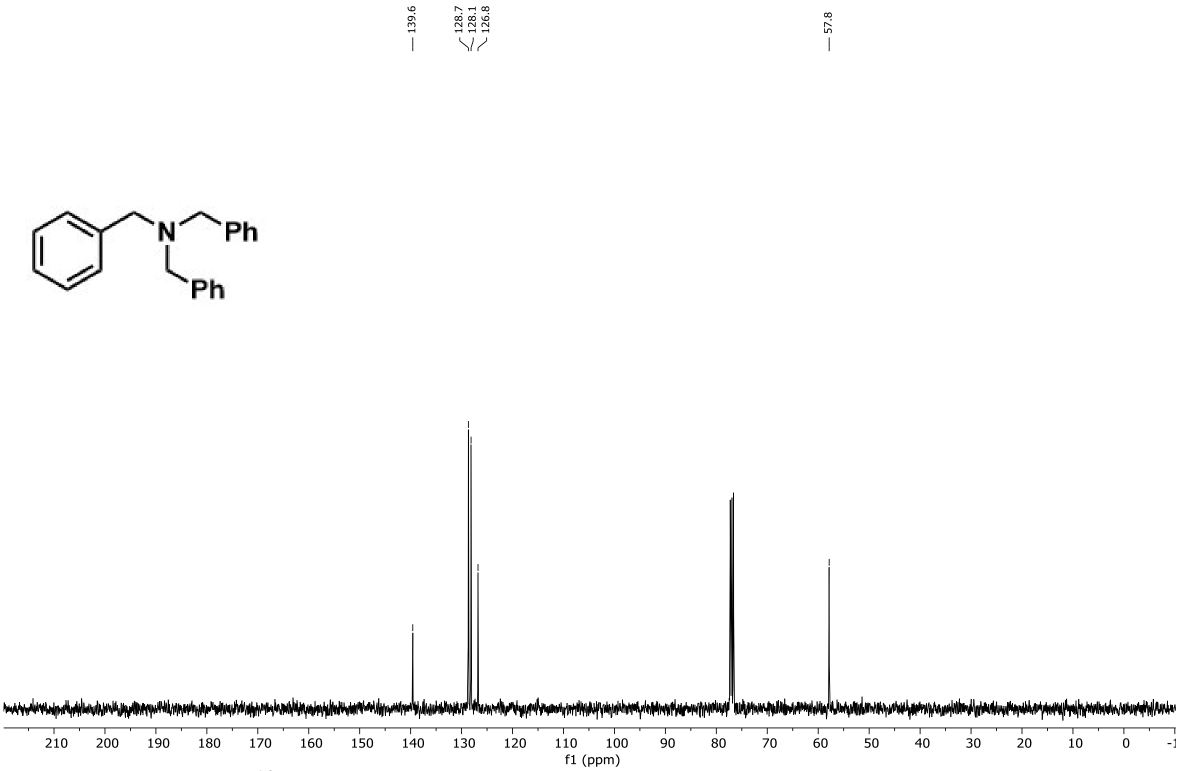

^{13}C NMR (101 MHz, $CDCl_3$) Tribenzylamine (**3h**)

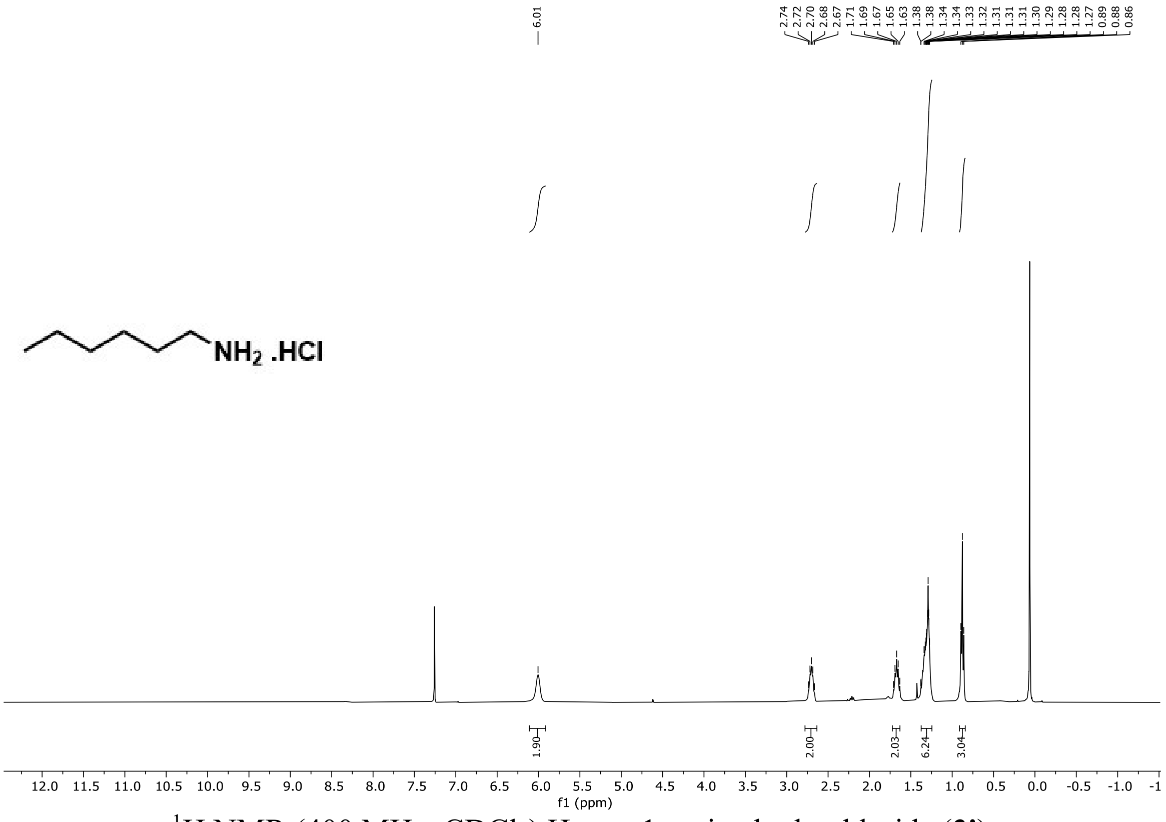

^{1}H NMR (400 MHz, $CDCl_3$) Hexan-1-amine hydrochloride (**3i**)

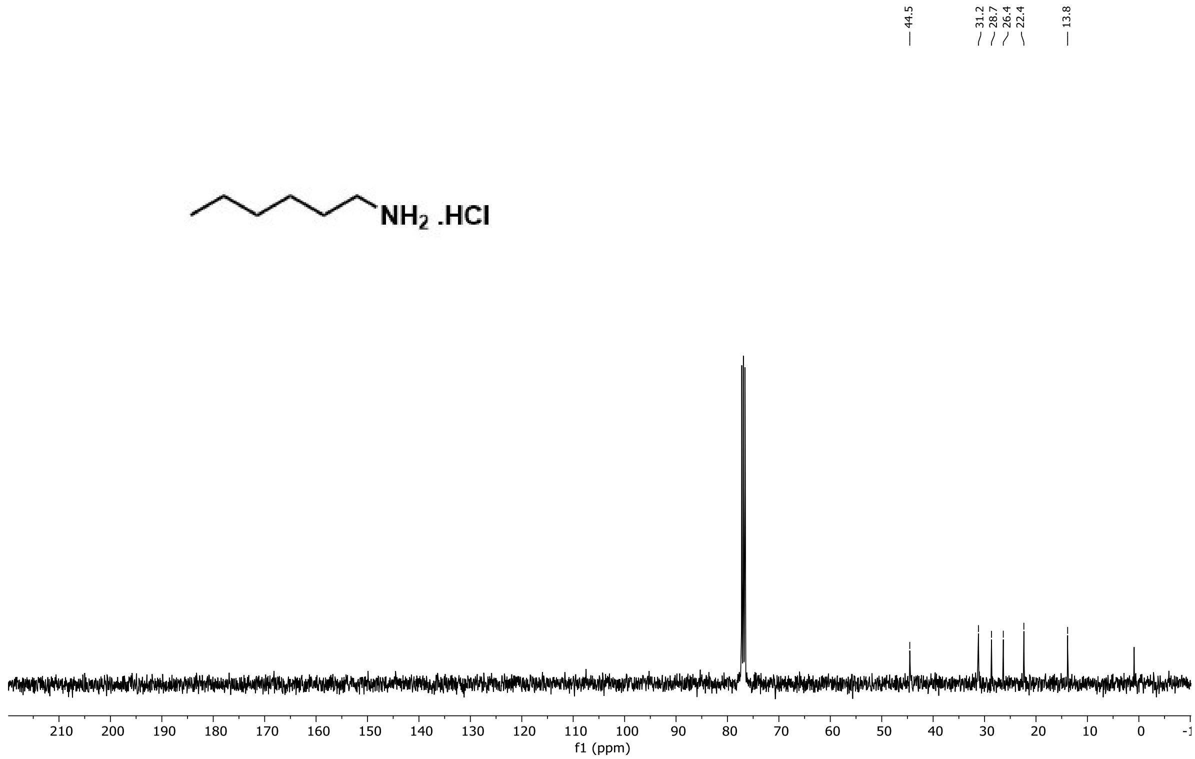

^{13}C NMR (101 MHz, $CDCl_3$) Hexan-1-amine hydrochloride (**3i**)

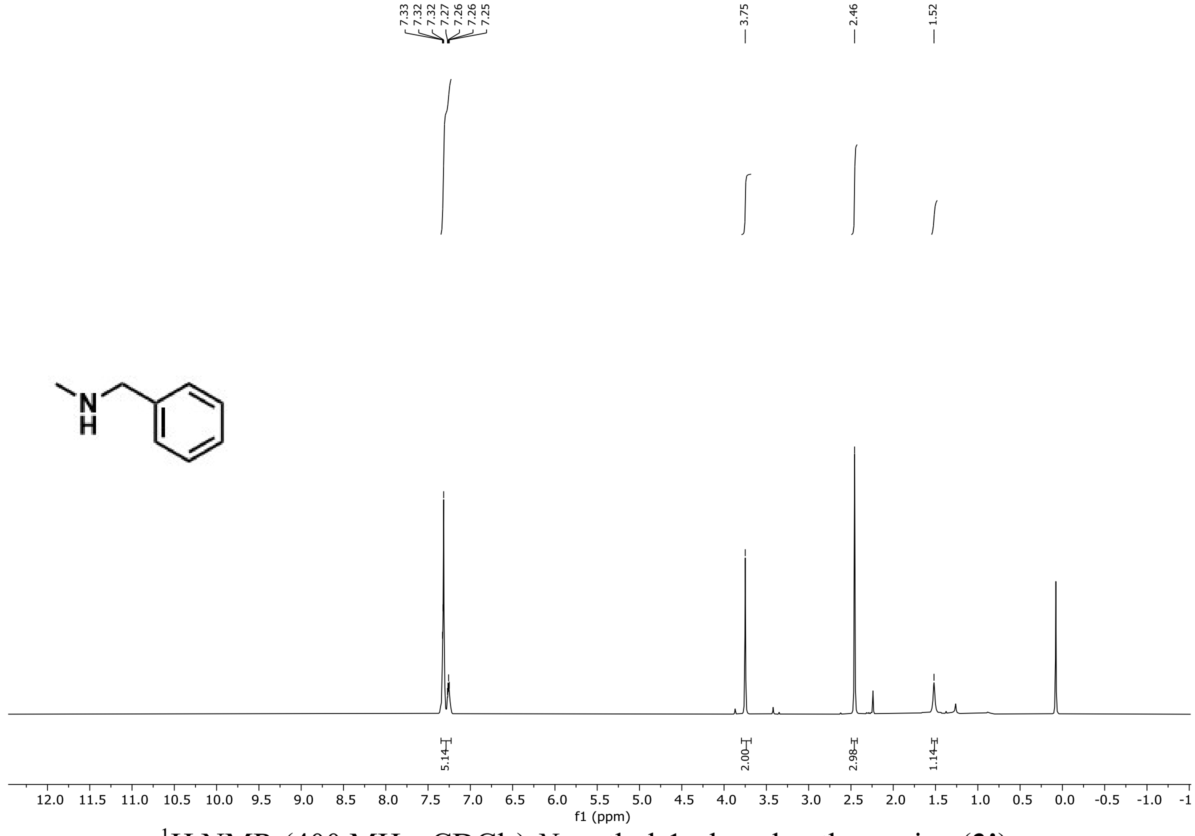

^{1}H NMR (400 MHz, $CDCl_3$) *N*-methyl-1-phenylmethanamine (**3j**)

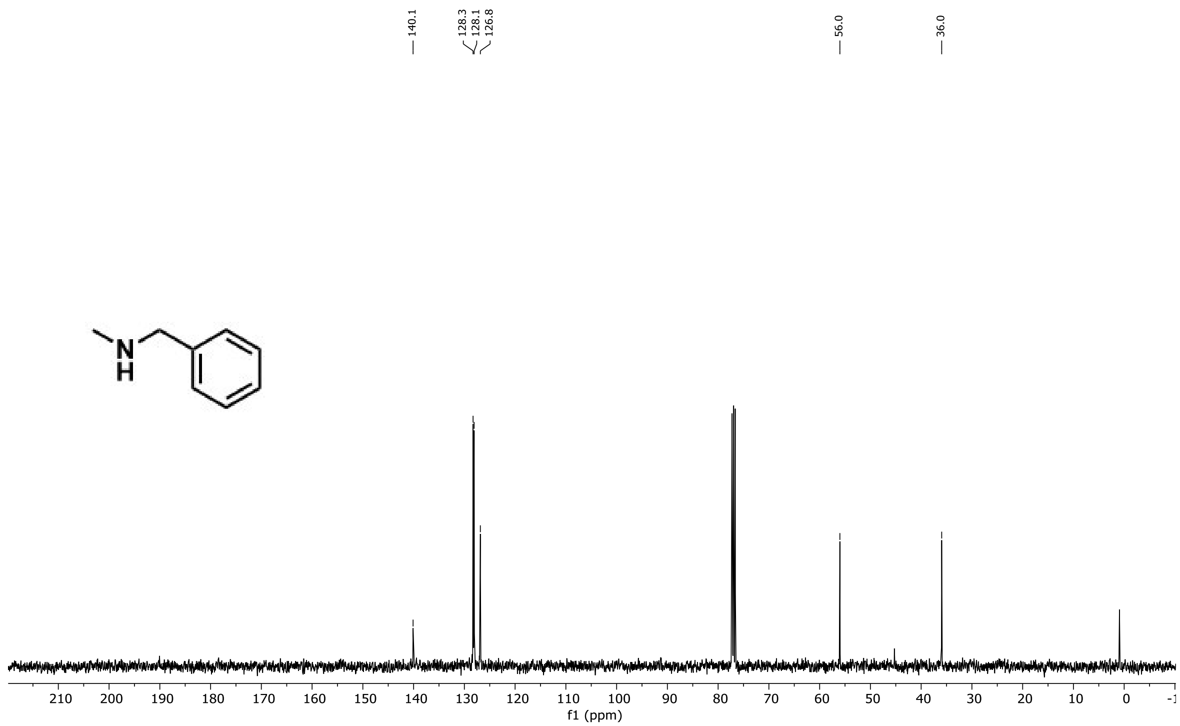

^{13}C NMR (101 MHz, $CDCl_3$) *N*-methyl-1-phenylmethanamine (**3j**)

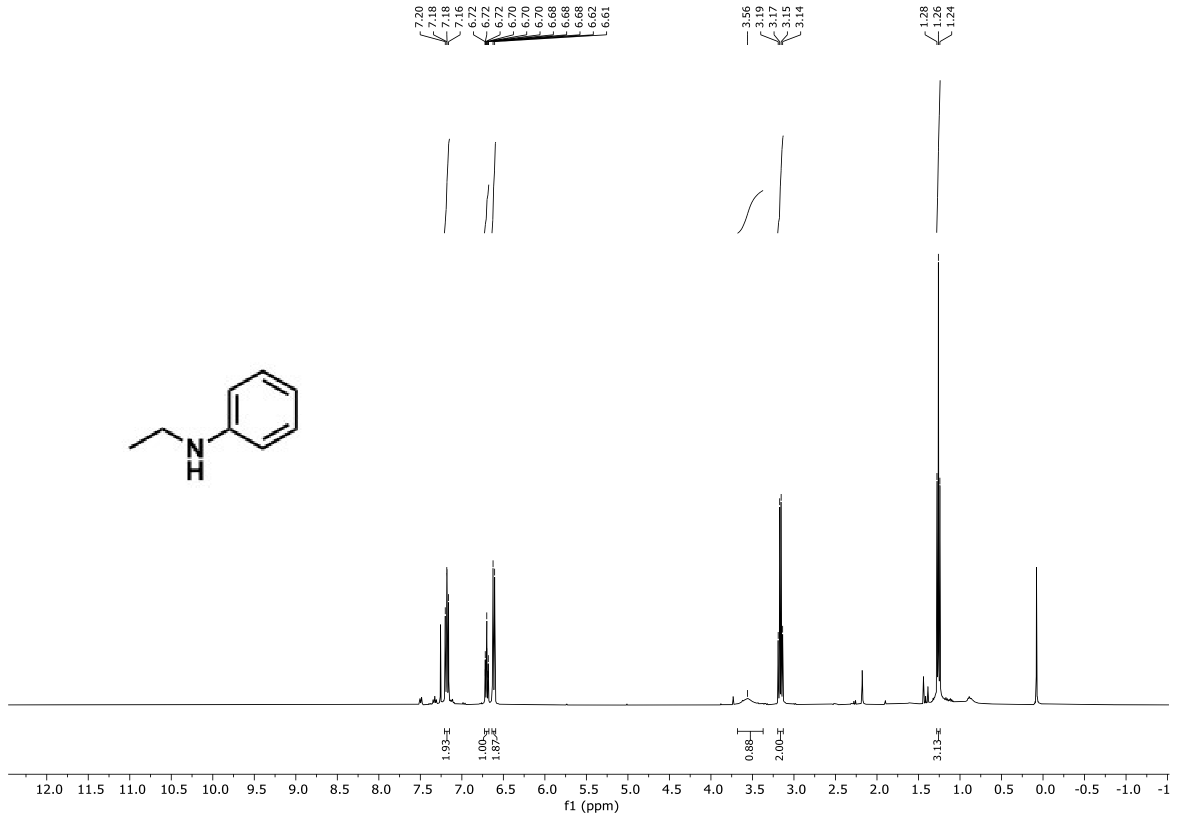

^{1}H NMR (400 MHz, $CDCl_3$) *N*-ethylaniline (**3k**)

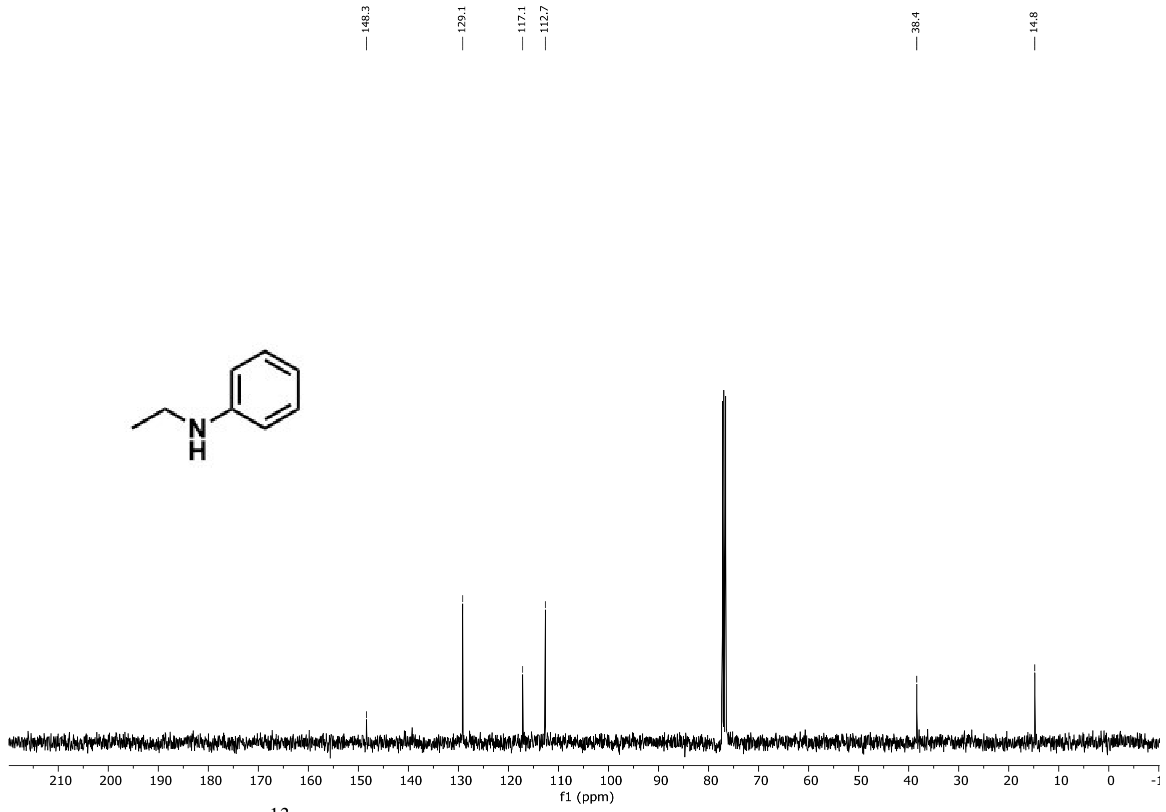

^{13}C NMR (101 MHz, $CDCl_3$) *N*-ethylaniline (**3k**)

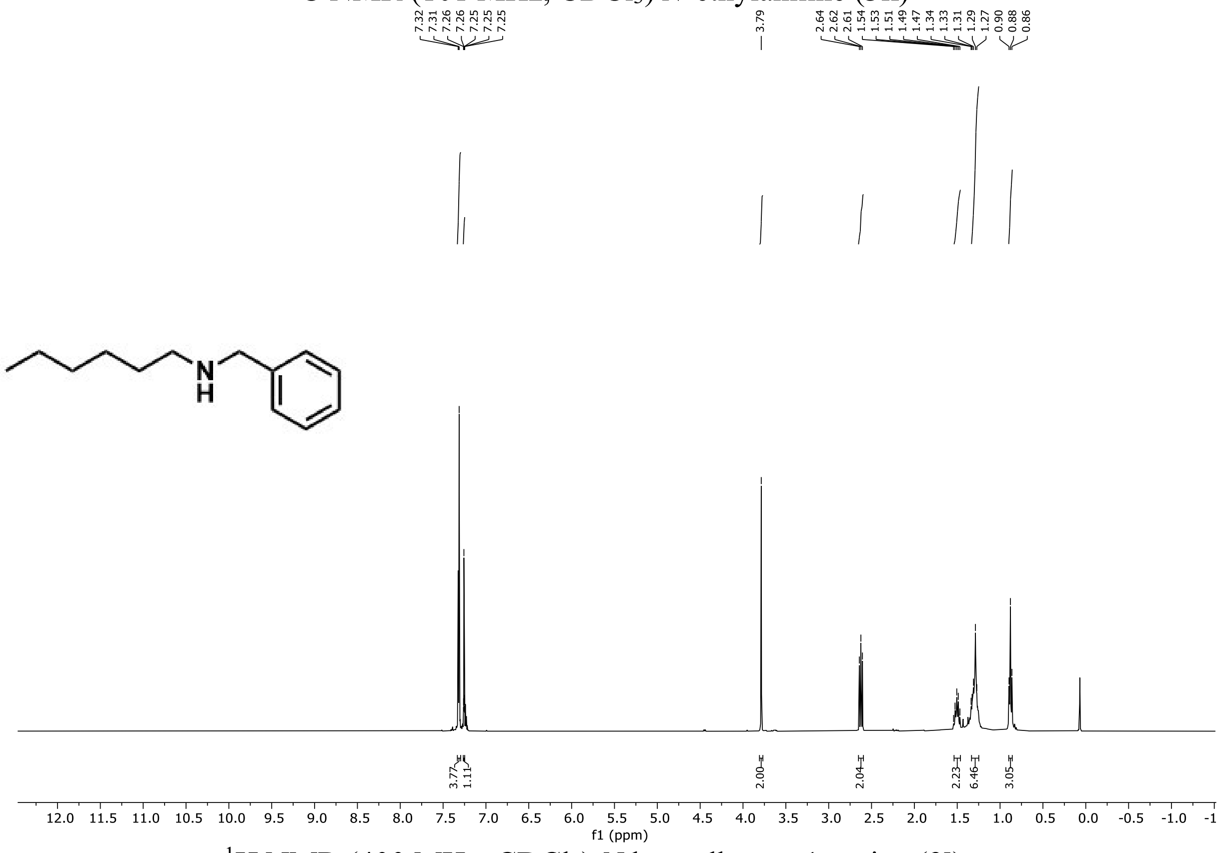

^{1}H NMR (400 MHz, $CDCl_3$) *N*-benzylhexan-1-amine (**3l**)

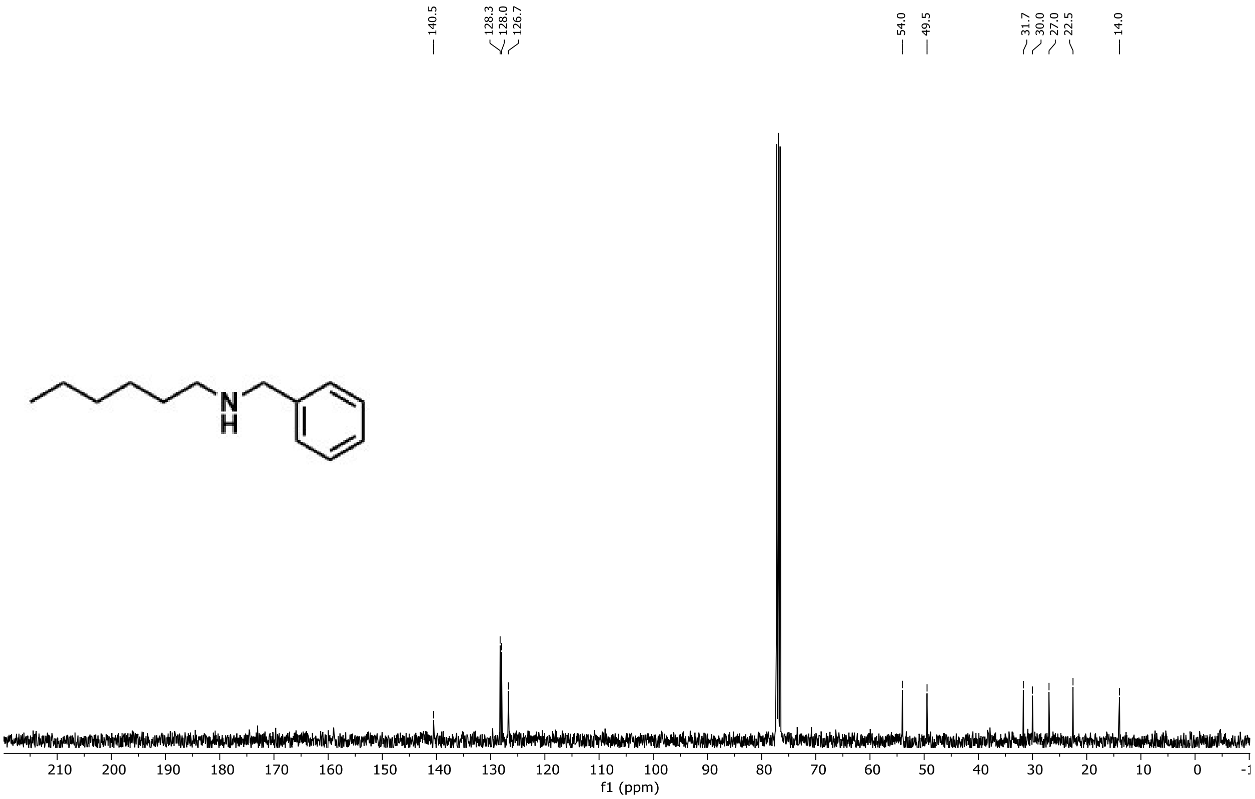

^{13}C NMR (101 MHz, $CDCl_3$) *N*-benzylhexan-1-amine (**3l**)

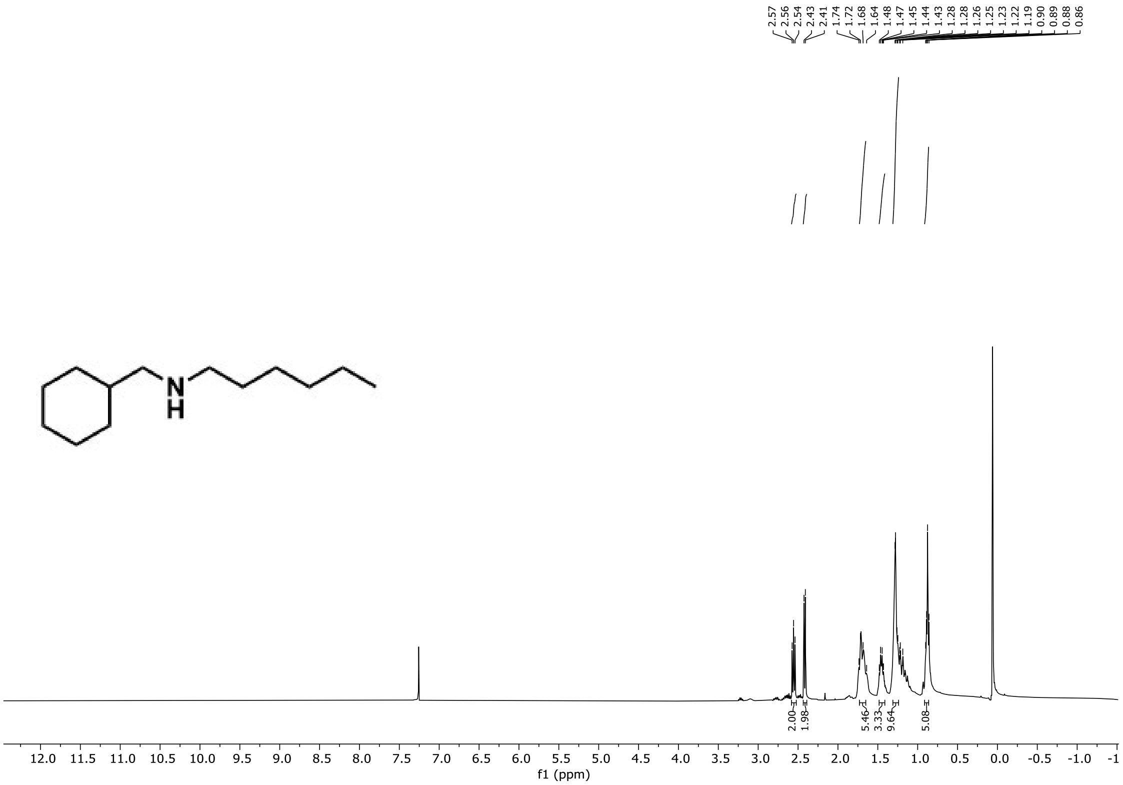

^{1}H NMR (400 MHz, $CDCl_3$) *N*-(cyclohexylmethyl)hexan-1-amine (**3m**)

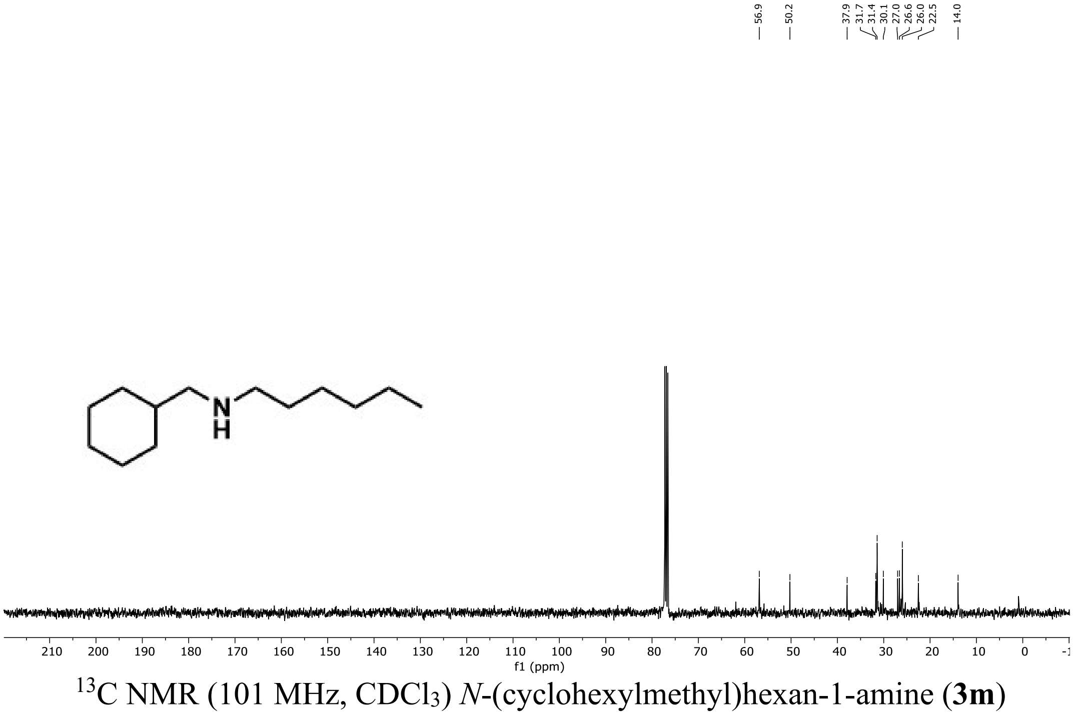

^{13}C NMR (101 MHz, $CDCl_3$) *N*-(cyclohexylmethyl)hexan-1-amine (**3m**)

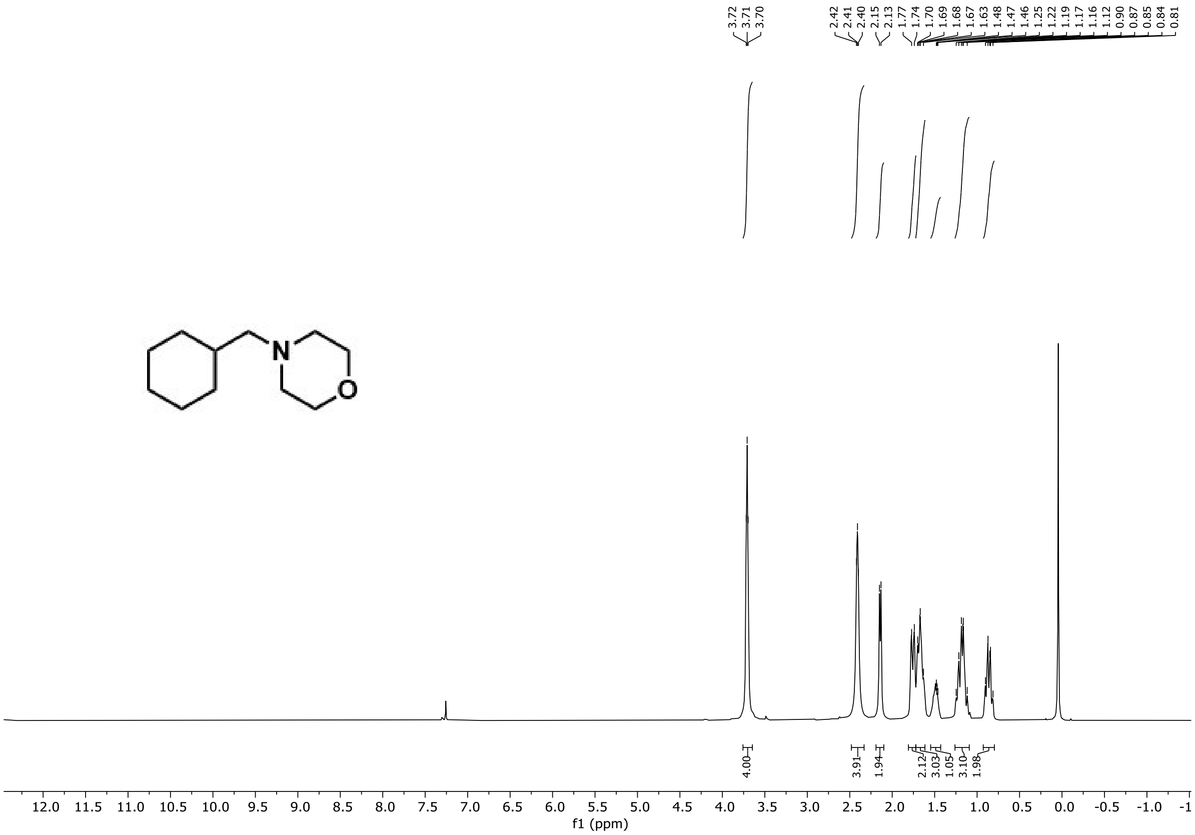

^{1}H NMR (400 MHz, $CDCl_3$) 4-(cyclohexylmethyl)morpholine (**3n**)

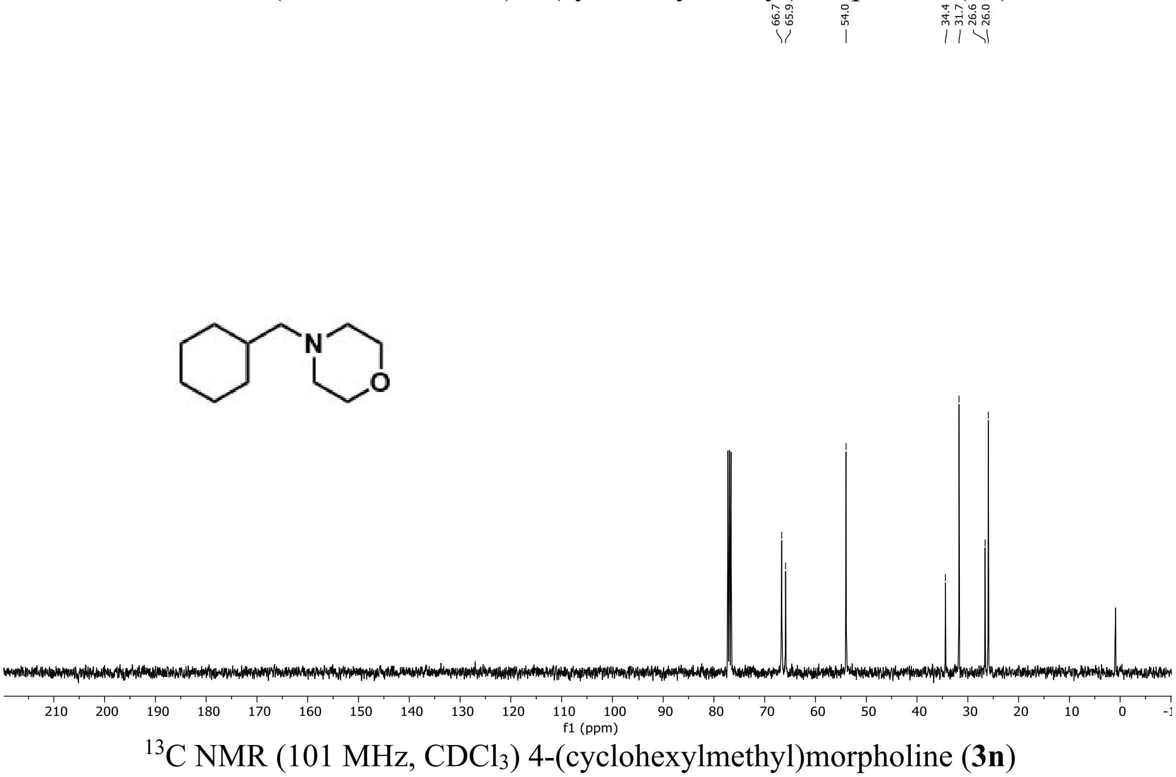

^{13}C NMR (101 MHz, $CDCl_3$) 4-(cyclohexylmethyl)morpholine (**3n**)

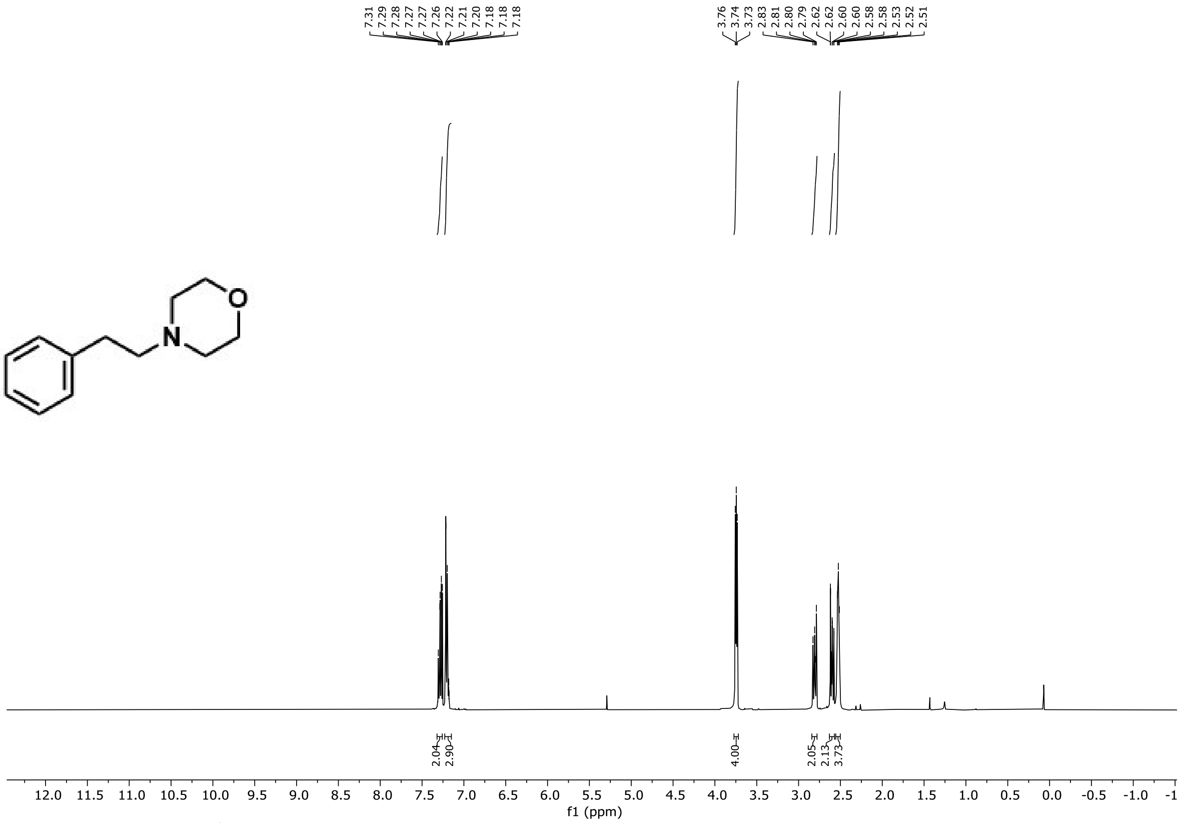

^{1}H NMR (400 MHz, $CDCl_3$) 4-phenethylmorpholine (**3o**)

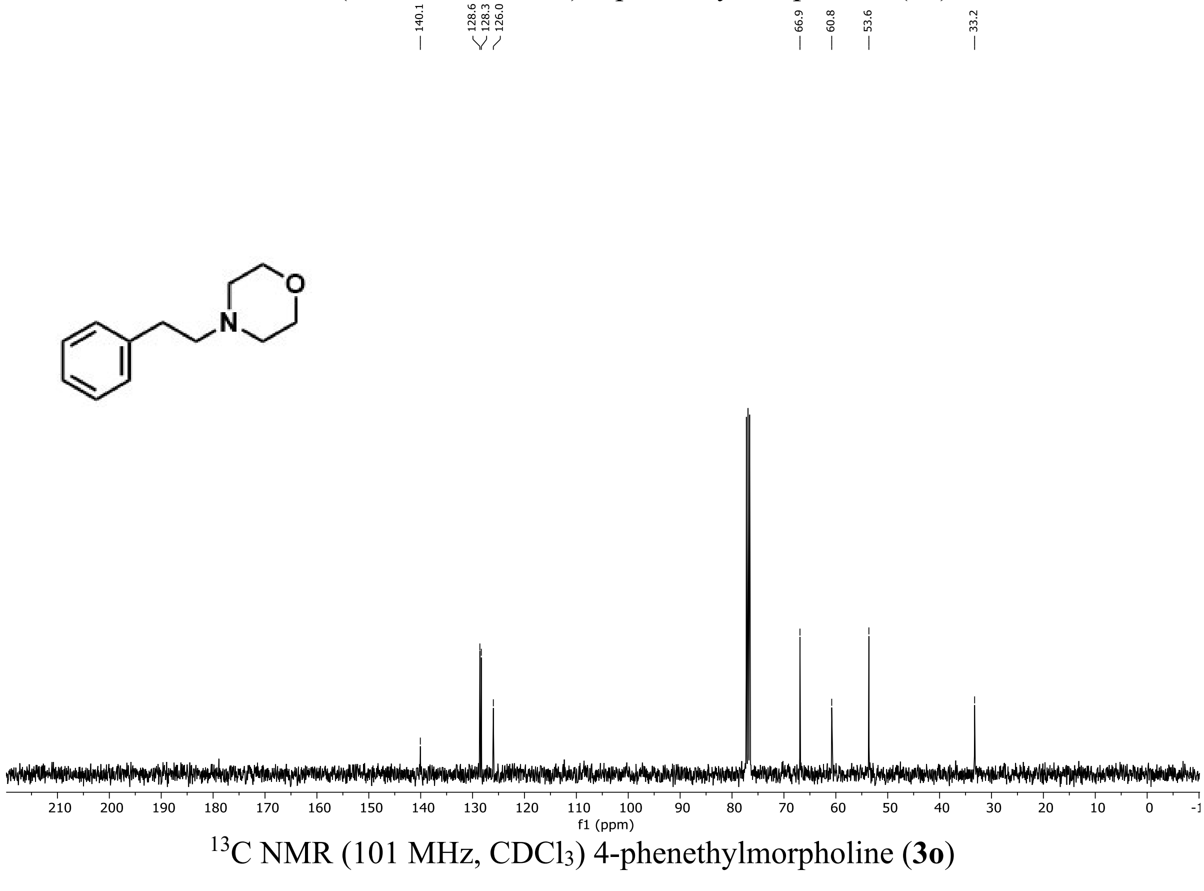

^{13}C NMR (101 MHz, $CDCl_3$) 4-phenethylmorpholine (**3o**)

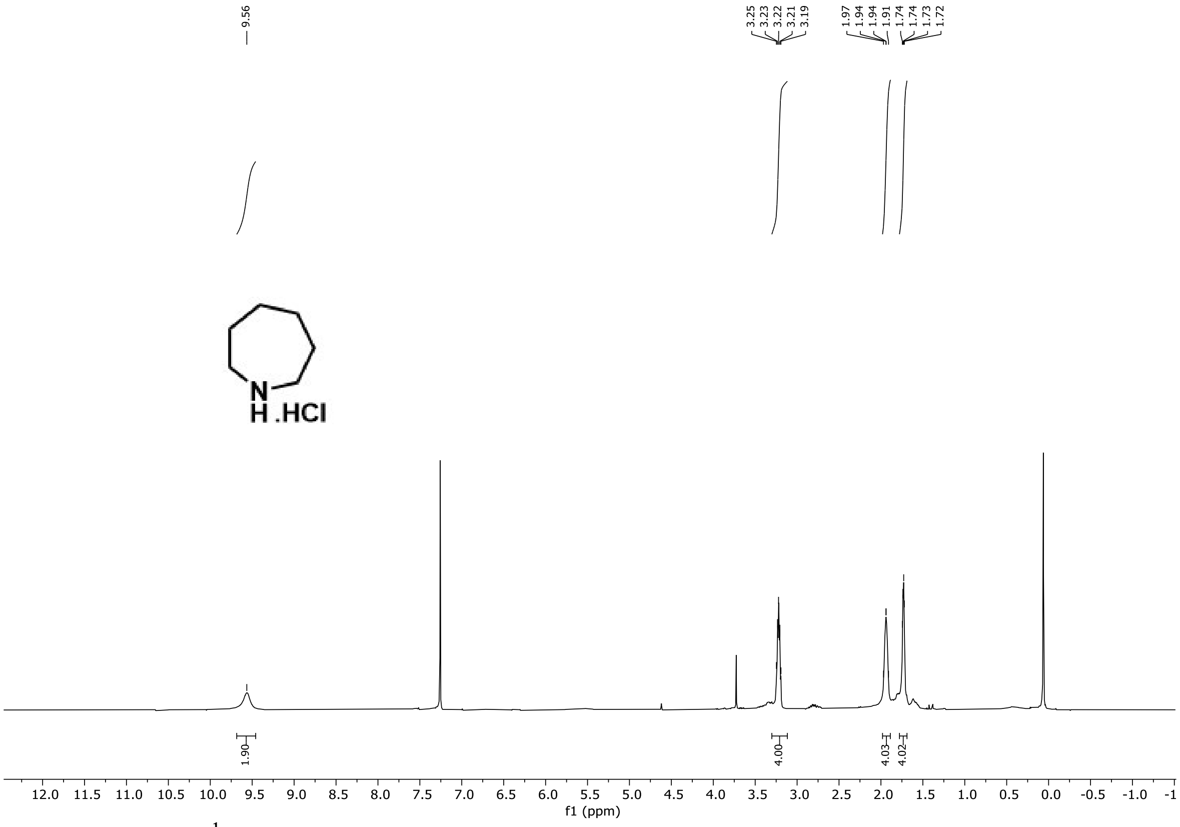

^{1}H NMR (400 MHz, $CDCl_3$) Azepane hydrochloride (**3p**)

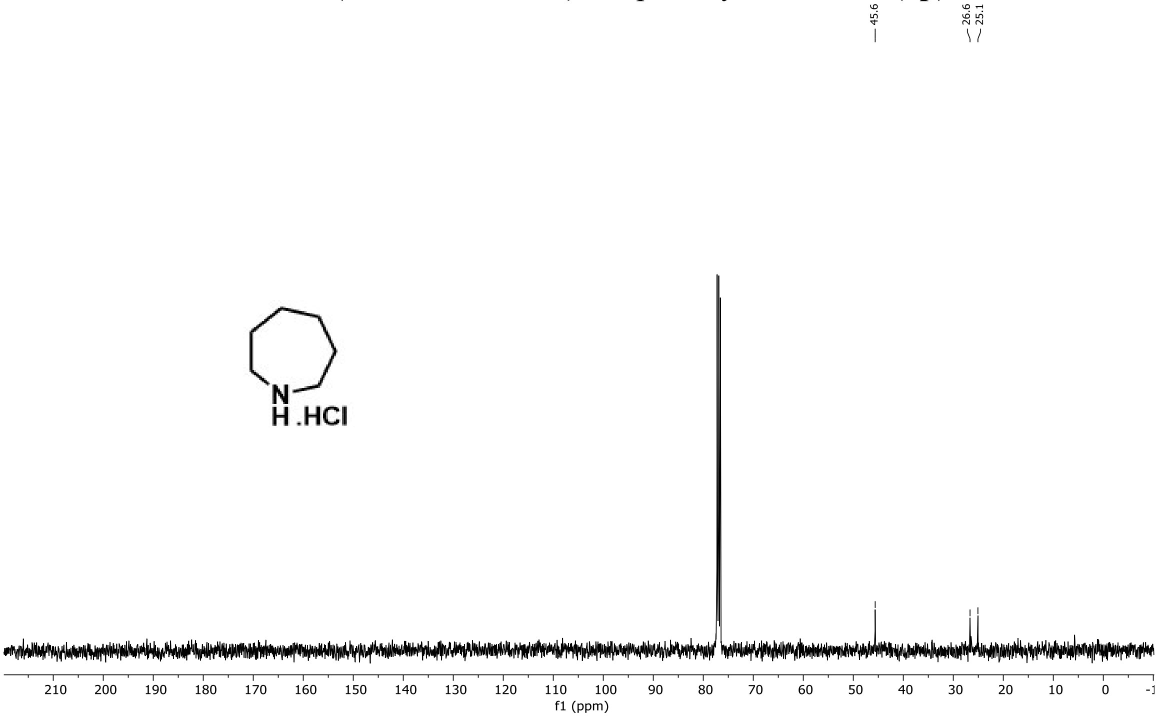

^{13}C NMR (101 MHz, $CDCl_3$) Azepane hydrochloride (**3p**)
